# A predictive model based on maternal immune-inflammatory markers for ARDS identification in mechanically ventilated late-preterm and term neonates

**DOI:** 10.3389/fimmu.2026.1889452

**Published:** 2026-10-06

**Authors:** Chao Zhang, Hui Jing, Dekai Xu, Zhixing Gao, Kangjin Diao, Xinmiao Yang, Yong Ji, Huaiqing Yin, Tongyan Han, Shirun Wu

**Affiliations:** 1 Department of Pediatric, Peking University Third Hospital, Beijing, China; 2 First Clinical Medical College of Shanxi Medical University, Taiyuan, China; 3 Department of Neonatal Intensive Care, First Hospital of Shanxi Medical University, Taiyuan, China; 4 Department of Pediatrics, Shanxi Medical University, Taiyuan, China; 5 Department of Neonatal Intensive Care, Shanxi Children's Hospital (Maternal and Child Health Hospital & Obstetrics and Gynecology Hospital), Taiyuan, China

**Keywords:** acute respiratory distress syndrome, immune‑inflammation index, machine learning, neonatal, pregnancy

## Abstract

**Objective:**

To develop and internally evaluate a model that uses maternal blood markers obtained before delivery to differentiate neonatal acute respiratory distress syndrome (ARDS) from other causes of respiratory distress in late-preterm and term neonates requiring mechanical ventilation.

**Methods:**

This retrospective study enrolled 227 mother-infant pairs from two tertiary hospitals between June 2024 and January 2026. Neonates born at 34–40 weeks’ gestation with ventilation-dependent respiratory distress were divided into a training cohort and an internal test cohort. Predictors were screened using elastic-net regression with 1,000 bootstrap resamples. Four feature sets and six algorithms were then compared by 20 repeats of stratified five-fold cross-validation, and a six-predictor random forest (RF) was ultimately selected. Discrimination, calibration and net benefit were assessed, with 500-resample bootstrap optimism correction and Shapley additive explanation (SHAP) interpretation.

**Results:**

Maternal systemic immune-inflammation index (SII) and related inflammatory ratios were higher, and antenatal infection was more frequent, in the ARDS group, whereas recorded antenatal corticosteroid (ACS) use was less frequent (P < 0.001). Six predictors were retained in the final model: SII, antenatal infection, maternal age, gestational hypothyroidism, gestational diabetes mellitus and ACS use. The principal internal performance estimate, the mean training-set cross-validated area under the curve (AUC) was 0.729 ± 0.080, the fixed-pipeline optimism-corrected AUC was 0.793 and the internal test-set AUC was 0.827 (95% CI, 0.712–0.923), with an average precision of 0.828 and a Brier score of 0.175. These three values are not interchangeable and are reported as one principal and two secondary estimates, and none represents fully independent performance. At the training-derived threshold of 0.424, sensitivity was 78.8% and specificity 77.8%. SHAP identified SII as the largest contributor, without implying a causal effect for any predictor. Logistic regression using the same six predictors reached a numerically higher internal test-set AUC (0.838 versus 0.827, ΔAUC = −0.011, P = 0.744) and a cross-validated AUC of 0.706 ± 0.082 versus 0.729 ± 0.080, the RF therefore conferred no discriminative advantage.

**Conclusions:**

In mechanical ventilation needed neonates born at 34–40 weeks' gestation with respiratory distress, the six-predictor RF showed moderate discrimination, whereas evidence of adequate calibration was limited. The model remains preliminary and requires external validation, recalibration and prospective clinical-impact evaluation before clinical use.

## Introduction

Neonatal acute respiratory distress syndrome (ARDS) is a severe form of lung injury that is often associated with adverse outcomes. Immature lung development and limited anti-inflammatory defenses place neonates at higher risk of respiratory disease. ARDS pathogenesis is closely associated with systemic inflammation and immune-mediated injury, but effective biomarkers for early prediction remain lacking. An international cohort of 15,916 hospitalized neonates reported an ARDS prevalence of 1.5%, with pneumonia, asphyxia and early-onset sepsis as the leading triggers (1). Most cases developed rapidly after birth, with cumulative incidences of 65.6%, 86.7%, and 94.1% within 24, 48 and 72 hours, respectively (1). The 2017 Montreux criteria are widely used to diagnose neonatal ARDS (2). Direct ARDS accounts for 51.5% of cases in term infants, whereas indirect ARDS is more common in preterm neonates and is often triggered by infections such as sepsis or necrotizing enterocolitis (3). These processes are closely linked to immune-mediated injury (4, 5).

Immune cells are central to ARDS pathogenesis. Alveolar macrophages recruit other immune cells to the lungs by releasing pro-inflammatory cytokines (6–8), whereas neutrophils damage lung tissue through neutrophil extracellular traps (NETs) (9, 10). Neonates with ARDS have elevated cell-free DNA and increased IL-6, TNF-α and MIP-1α, suggesting that inflammatory markers may predict ARDS incidence and severity (11, 12). The systemic immune-inflammatory index (SII) and systemic inflammatory response index (SIRI), composite indices calculated from combinations of neutrophil, platelet, lymphocyte and monocyte counts, have been studied in malignancies (13–15) and cerebrovascular diseases (16). In neonates, SII and SIRI have been identified as independent risk factors for respiratory distress syndrome (17, 18), and our previous study found that early SIRI in premature infants was associated with ARDS occurrence (19). Maternal immune-inflammatory status during late pregnancy may influence the intrauterine environment and fetal lung development (20). Maternal systemic inflammatory indices have also been associated with fetal growth, including birth weight, in physiological pregnancies (21). However, the predictive value of maternal markers for neonatal ARDS has not been well characterized.

A model based on maternal markers may help distinguish ARDS from other causes of respiratory distress within a high-risk population of mechanically ventilated neonates (22). We therefore combined maternal immune-inflammatory indices obtained within one week before delivery with routinely recorded obstetric variables, compared six machine-learning algorithms and internally evaluated the resulting model for risk stratification in this selected population.

## Materials and methods

### Study design

We conducted a retrospective observational study using data from neonates and their mothers admitted to the NICUs of two tertiary Grade A hospitals between June 1, 2024 and January 1, 2026. Eligible neonates were born at 34–40 weeks of gestation, developed respiratory distress within 7 days after birth and required mechanical ventilation. The lower gestational bound of 34 weeks was set because respiratory failure below this age is dominated by surfactant-deficient respiratory distress syndrome, whose mechanisms differ from those of ARDS and which the Montreux criteria require to be excluded. The upper bound of 40 weeks excluded post-term pregnancies, in which placental ageing introduces a different inflammatory context. The 7-day window corresponds to the acute-onset requirement of the Montreux definition, and mechanical ventilation was required because oxygenation index, which the definition uses to grade severity, can be computed only in ventilated neonates. Stratified random sampling was used to divided the cohort into a training set (n = 158) and an internal test set (n = 69) at a 7:3 ratio. The cohort flow is summarized in **Figure 1**. Predictor screening, candidate-set construction, final model selection and operating-threshold derivation were performed in the training set. The same internal test partition had been accessed during earlier exploratory evaluation of the initial eight-variable model and RF permutation importance. It was therefore not a strictly untouched holdout. The final six-predictor RF model and its operating thresholds were selected without using internal test-set performance. For this retrospective analysis, the intended prediction time was immediately before delivery, at which point all six model inputs, including recorded antenatal corticosteroid (ACS) use, were ascertainable and neonatal outcomes were not yet known. The model was not evaluated for use at the earlier time of blood sampling and was not intended to guide the initial decision to administer ACS. The analysis addressed discrimination within this selected neonatal respiratory-distress cohort and was not intended as a screening tool for an unselected antenatal population.

**Figure 1:**
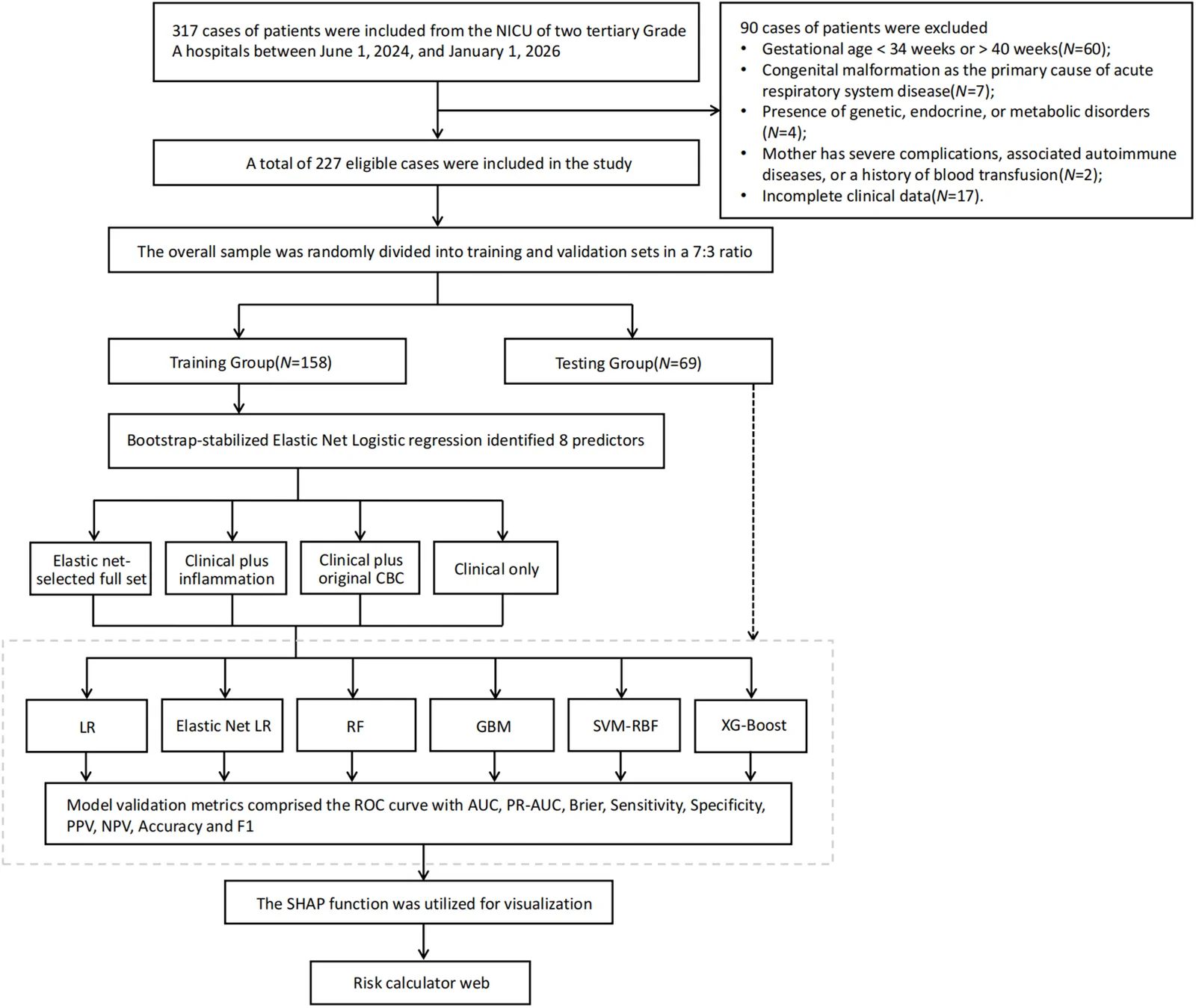
The flowchart of the study. ARDS, acute respiratory distress syndrome; CBC, Complete Blood Count; LR, Logistic Regression; Elastic Net LR, Elastic Net Logistic Regression; RF, Random Forest; GBM, Gradient Boosting Machine; SVM-RBF, Support Vector Machine with Radial Basis Function kernel; XG-Boost, extreme gradient boosting; ROC, Receiver Operating Characteristic; AUC, Area Under the Curve; AP, average precision; Brier, Brier Score; PPV, Positive Predictive Value; NPV, Negative Predictive Value; F1, F1 Score; SHAP, Shapley additive explanations.

Study design, analysis and reporting were guided by TRIPOD+AI recommendations (23). The analysis followed a documented workflow in which final model selection and threshold derivation used the training set.

### Data collection and definitions

We extracted gestational age, ARDS status, maternal pregnancy complications and the final prenatal complete blood count, obtained 3–7 days before delivery, from electronic medical records. ACS was not routinely administered in this cohort. ACS could be administered on an individual basis under the Queensland Clinical Guidelines: Antenatal Corticosteroids (24). This applied to women between 34 and 36+6 weeks of gestation at administration when preterm delivery was considered imminent. Indications were evaluated comprehensively and included gestational diabetes mellitus, gestational hypertension and fetal growth restriction. ACS was initiated only after counselling and informed consent had been obtained from the woman and her family. The regimen was intramuscular dexamethasone 6 mg every 12 h for four doses. Dates of blood sampling and ACS initiation were cross-checked against the original medical records. In all ACS recipients, the blood sample used to calculate the inflammatory indices had been obtained at least 72 h before the first ACS dose. An acute corticosteroid effect on the measured blood-cell counts was therefore unlikely. The interval between ACS administration and delivery and the completeness of the administered course were not systematically recorded. The primary indication in each individual case was also not systematically recorded. These details could not be analyzed. Neonatal ARDS was diagnosed according to the 2017 Montreux criteria. Diagnosis required acute respiratory manifestations within one week and bilateral diffuse abnormalities on lung imaging. RDS, TTN and congenital anomalies were excluded before diagnosis. Cardiac-derived edema was excluded by echocardiography. Each case was adjudicated independently by three neonatologists. They reviewed the clinical course, oxygenation data and lung imaging. All three were blinded to the maternal complete blood count and to every derived immune-inflammatory index. Thus outcome ascertainment could not have been influenced by the candidate predictors. Where the three assessments did not agree, the case was resolved by consensus discussion among the three adjudicators. A case was classified as ARDS only once all three agreed. Severity was classified by oxygenation index as mild (4–8), moderate (8–16) or severe (>16). Full diagnostic criteria are provided in **Supplementary Appendix 1**. NRDS, TTN and infectious pneumonia were diagnosed according to the relevant guidelines. Their differential criteria are provided in **Supplementary Appendix 2**. Recorded maternal conditions included gestational diabetes mellitus, gestational hypertension, chorioamnionitis, gestational hypothyroidism and placenta previa. We also recorded premature rupture of membranes, antenatal infection within 14 days before delivery, fetal distress and ACS use. Antenatal infection was defined as a maternal bacterial, viral or other systemic infection diagnosed by a healthcare institution within 14 days before delivery. Diagnostic criteria for the maternal conditions are provided in **Supplementary Appendix 3**. The composite inflammatory indices were:

NLR:


$$neutrophil-to-lymphocyte\;ratio=\frac{absolute\;neutrophil\;count}{absolute\;lymphocyte\;count}$$

PLR:


$$blood\;platelet-to-lymphocyte\;ratio=\frac{platelet\;count}{absolute\;lymphocyte\;count}$$

SII:


$$systemic\;immune-inflammation\;index=\frac{absolute\;neutrophil\;count\;\times \;platelet\;count}{absolute\;lymphocyte\;count}$$

SIRI:


$$systemic\;inflammation\;response\;index=\frac{absolute\;neutrophil\;count\;\;\times \;absolute\;monocyte\;count}{absolute\;lymphocyte\;count}$$

### Patient inclusion and study groups

Neonates: Inclusion criteria were as follows: (1) Gestational age ≥ 34 weeks and ≤ 40 weeks; (2) Respiratory distress began within 7 days after birth and required mechanical ventilation. Exclusion criteria were as follows: (1) Gestational age < 34 weeks or > 40 weeks; (2) Major congenital malformations as the primary cause of acute respiratory disease, including congenital heart disease; (3) Genetic, endocrine, or metabolic diseases. (4) Incomplete clinical data.

Mothers were eligible if they met the following criteria: (1) Gestational age at delivery met the neonatal inclusion criteria. (2) A prenatal complete blood count obtained 3–7 days before delivery was available. (3) Clinical data were complete. Exclusion criteria were as follows: (1) A maternal hematological disorder, recent blood transfusion or medication use that could independently affect the prenatal complete blood count. (2) Acute severe antenatal infection or active autoimmune disease that could directly cause neonatal ARDS.

The ARDS group included infants who met the Montreux criteria for neonatal ARDS. This included infants with concurrent RDS, TTN, pneumonia or other respiratory conditions. The non-ARDS group included infants with isolated RDS, TTN, pneumonia or other respiratory conditions who did not meet the ARDS criteria.

### Sample size estimation

Sample size was estimated using the events-per-variable (EPV) ratio. We planned to include five to six predictors and set the EPV at 10. The resulting minimum sample size was approximately 200, consistent with previous studies of similar scope (22). The final six-predictor model had approximately 12 events per variable in the training set. This met the conventional EPV threshold of 10. However, more recent sample-size criteria for prediction models would require larger samples. Predictor screening, comparison of the six candidate algorithms and SHAP interpretation were all performed within the training set. At this sample size, these procedures were exploratory rather than confirmatory. Estimates of variable importance were therefore unstable. The internal test set contains only 33 events. Estimates from this set were imprecise, as reflected in the wide confidence intervals. 


$$\text{Total sample size}=\frac{EPV\;\times \;number\;of\;predictor\;variables}{event\;incidence}$$

### Data preprocessing and quality control

Before modeling, we checked variable types, ranges, duplicate records, missingness and logical consistency. We recalculated NLR, PLR, SII and SIRI from the source blood-count variables and compared them with the recorded values. Values flagged by range or consistency checks were verified against the original laboratory or medical records. Continuous variables were imputed with training-set medians and binary variables with training-set modes. During cross-validation, imputation and standardization were fitted within each training fold and applied only to the corresponding validation fold. The internal test set was transformed using parameters estimated from the full training set. Missingness among the principal candidate variables was low, and no substantial case exclusions resulted from missing data.

Continuous variables were presented as medians with interquartile ranges, and categorical variables as counts with percentages. Continuous variables were compared between the ARDS and non-ARDS groups using the Mann–Whitney U test. Categorical variables were compared using the χ² test or Fisher’s exact test. Baseline balance between the training and internal test sets was assessed descriptively using standardized mean differences rather than significance testing. Standardized mean difference for every candidate predictor and for the outcome is reported in **Supplementary Table S5**.

### Variable screening and candidate set construction

Among 24 recorded non-outcome variables, low birth weight was excluded *a priori* because it is determined after birth and was unavailable at the intended prenatal prediction time. The remaining 23 eligible candidate predictors were screened only in the training set. The complete list is provided in **Supplementary Appendix 4**. We used bootstrap-stabilized elastic-net logistic regression to improve selection stability in this small sample and address correlated blood-cell indicators (**Supplementary Figure S1**). We drew 1,000 bootstrap samples with replacement and selected penalty parameters within each sample using stratified cross-validation. Candidate l1_ratio values were 0.1, 0.3, 0.5, 0.7, 0.9 and 1.0, and C was searched on a logarithmic grid using ROC-AUC as the optimization criterion. For each repetition, we recorded whether each variable was selected and the sign and magnitude of its standardized coefficient.

The study-specific empirical retention criteria required a bootstrap inclusion frequency of at least 0.70 and a dominant-sign frequency of at least 0.75. We also required an absolute median standardized coefficient of at least 0.03. The retained set was limited to 4–8 predictors to constrain model complexity in this dataset. These cut-offs were pragmatic study-specific rules and were not treated as literature-derived standards.

### Candidate variable set construction and clinical redundancy reduction

We constructed candidate variable sets from the initial elastic-net results. We then reduced clinical redundancy to avoid repeated representation of correlated or formula-derived information. The full screened set comprised maternal age (MA), gestational diabetes mellitus (GDM), gestational hypothyroidism (GH), antenatal infection within 14 days before delivery (AI), PLT, ACS, SII, and NEU. Because SII is derived from PLT, NEU and LYM, retaining SII together with its components could increase redundancy, model complexity and overfitting. Clinical plus Inflammation group therefore retained SII as the composite inflammatory indicator and excluded PLT and NEU. The resulting set comprised AI, ACS, MA, GH, GDM and SII. The composite inflammatory indices were strongly inter-correlated in the training set, as expected from their shared derivation from the same blood-cell counts. SII correlated with PLR (Spearman ρ = 0.807), NLR (0.775), SIRI (0.619), PLT (0.594) and NEU (0.589), and WBC correlated with NEU (0.959), and the full matrix is shown in **Supplementary Figure S1**. Elastic-net regularization is designed to handle correlated blocks of variables such as these. Among the composite indices, only SII met all three stability criteria. Its selection frequency was 0.852, direction stability was 0.834 and absolute standardized median coefficient was 0.216. PLR, SIRI and NLR did not meet all three criteria, with direction stability of 0.513, 0.455 and 0.424, respectively.

Four variable sets were constructed after bootstrap screening: (1) Clinical plus Inflammation (Group 1), which added SII to the clinical variables. (2) Selected (Group 2) contained the full eight-variable screened set. (3) Clinical only (Group 3) contained the clinical variables. (4) Clinical plus CBC (Group 4) added PLT and NEU to the clinical variables. All four sets were compared within the training set without using internal test-set performance.

### Candidate algorithms, model training, and model selection

Model training and candidate model comparison were performed in the training set using repeated stratified 5-fold cross-validation with 20 repeats. Candidate algorithms included Logistic Regression, Elastic Net Logistic Regression, Random Forest (RF), Gradient Boosting Machine (GBM), XGBoost, and Support Vector Machine with Radial Basis Function kernel (SVM-RBF). The finalized configuration of each of the six candidate algorithms, including all hyperparameter values and the internal tuning grid used for elastic-net logistic regression, is reported in **Supplementary Table S6**. Logistic Regression and Elastic Net Logistic Regression served as benchmark models to evaluate the incremental value of nonlinear machine learning algorithms over traditional regression models.

Logistic Regression, Elastic Net Logistic Regression, and SVM-RBF are sensitive to variable scales. For these models, continuous variables were Z-score standardized within each training fold. The same standardization parameters were applied to the corresponding validation fold. Tree-based models used raw numerical variables. Machine learning pipelines contained all imputation, standardization, model fitting, and prediction steps. Each pipeline was fitted independently within each cross-validation fold to prevent information leakage between the training and validation folds.

Mean cross-validated ROC-AUC in the training set was the principal model-selection criterion. Algorithm–feature-set combinations were eligible if their mean AUC was within a study-specific tolerance of 0.01 below the maximum. Eligible combinations were ranked first by fewer predictors, then by lower mean cross-validated Brier score. A fixed algorithm-priority rule and higher mean AUC were the next ranking criteria, in that order. The priority rule did not affect the observed selection because the six-predictor RF had the highest mean cross-validated AUC. Calibration and decision-curve results from the internal test set did not enter the ranking. The final RF used 500 trees with no maximum depth constraint and a minimum leaf size of five. It used square-root feature sampling and a random seed of 42.

### Model performance evaluation

After selection, the locked RF pipeline was refitted using the full training set and assessed in the internal test set. Discrimination was summarized using ROC-AUC and average precision (AP). The prevalence of ARDS in the internal test set reported alongside AP. ROC-AUC, AP and Brier-score point estimates were calculated from the original internal test set. 95% percentile confidence intervals were obtained from 1,000 bootstrap resamples of that set.

Probabilistic prediction accuracy was evaluated using the Brier score, where a lower Brier score indicates a smaller mean squared error between predicted probabilities and actual outcomes. Model calibration was assessed via calibration curves, calibration intercept, and calibration slope. Calibration curves were plotted using a 4-bin grouping method based on predicted probability quantiles. We compared the mean predicted probability and actual ARDS incidence in each bin. Calibration intercept and slope were obtained by fitting a calibration model with the actual outcome as the dependent variable and the logit-transformed predicted probability as the independent variable, with ideal values of 0 and 1, respectively.

Operating thresholds were derived solely from out-of-fold predictions in the training set. These predictions were generated using repeated stratified five-fold cross-validation with ten repeats. The threshold maximizing Youden’ s index was used as the primary reporting threshold. Youden’s index gives equal weight to sensitivity and specificity, which is a statistical convention rather than a clinical cost-benefit judgement. A false negative may delay recognition of evolving ARDS. A false positive may prompt unnecessary escalation of monitoring or treatment. These consequences are not equivalent, and no locally validated estimates of their relative cost were available to us. We therefore report the Youden threshold only as a reference operating point. Sensitivity-oriented thresholds and the conventional fixed 0.50 threshold are reported as supplementary operating points (**Supplementary Table S1**). These alternatives allow a threshold matched to the relative harms of the two errors to be selected in any future application. These thresholds were locked and applied unchanged to the internal test set. Decision curve analysis described net benefit across threshold probabilities from 0.01 to 0.80, using treat-all and treat-none strategies as references. Decision curves were interpreted descriptively rather than as evidence of uniform clinical superiority.

### Internal validation, optimism correction, and benchmark comparison

A separate 500-repetition bootstrap analysis estimated optimism for the fixed final pipeline in the training set. In each repetition, we refitted the RF model in a bootstrap sample using the same six predictors and hyperparameters. We calculated performance in both the bootstrap sample and the original training set using the fitted model. The difference between these performance estimates provided an estimate of optimism. Mean optimism was subtracted from the apparent training-set AUC and Brier score. Predictor screening, feature-set construction and algorithm selection were not repeated within bootstrap samples.

After RF had been selected from training-set cross-validation, logistic regression and elastic-net logistic regression were fitted with the same six predictors as post-selection linear benchmarks. Their internal test-set ROC-AUC, AP, Brier score, calibration slope and decision curves were compared descriptively with those of RF. Paired bootstrap resampling of the internal test set estimated AUC differences and 95% percentile intervals. These benchmark comparisons did not determine primary-model selection.

### Model interpretation

We used SHapley Additive exPlanations (SHAP) to interpret predictions from the final RF model. Mean absolute SHAP values summarized each predictor’s overall contribution to model output. A beeswarm plot showed the distribution and direction of these contributions across participants. For binary variables, higher values indicated presence and lower values indicated absence.

Because SII had the largest global contribution, we generated a SHAP dependence plot to show how its values related to model output. We also generated partial-dependence and individual conditional-expectation curves to describe the marginal association between SII and predicted ARDS probability. The curves also showed how this association varied between participants. These plots described model behavior and were not used to define clinical diagnostic thresholds.

We selected illustrative correctly classified low-risk non-ARDS and high-risk ARDS cases from the internal test set for individual SHAP waterfall plots. These plots showed how each predictor shifted the estimated risk above or below the model’s baseline probability. The individual explanations illustrated how predictions were generated and were not interpreted causally.

### *Post hoc* sensitivity analysis according to ACS use

After model development, we conducted a *post hoc* exploratory sensitivity analysis in the full cohort. We examined whether recorded ACS use distorted or modified the association between SII and neonatal ARDS. This analysis assessed association only and did not inform predictor screening, model selection, hyperparameter tuning or threshold selection. It was also not used to estimate model performance. Because SII was right-skewed, it was log2-transformed and then standardized to a mean of 0 and a standard deviation of 1. We first fitted a multivariable logistic regression model including log2-transformed SII, ACS use, antenatal infection, maternal age, gestational hypothyroidism and gestational diabetes mellitus. An SII-by-ACS interaction term was then added to test for statistical evidence of effect modification. SII distributions were summarized using medians and interquartile ranges and compared using two-sided Mann–Whitney U tests. Associations are reported as odds ratios (ORs) with 95% confidence intervals (CIs).

## Results

### Participant characteristics

The study included 227 enrolled pairs comprised 107 ARDS and 120 non-ARDS neonates, distributed as 74 ARDS among 158 neonates in the training set and 33 ARDS among 69 neonates in the internal test set. Median maternal age was slightly higher in the ARDS group (P = 0.032). Gestational age, gender, low birth weight and most pregnancy complications did not differ significantly between groups (all P > 0.05). ACS use was more frequent in the non-ARDS group, whereas antenatal infection was more frequent in the ARDS group (both P < 0.001).

White blood cell, platelet, neutrophil and monocyte levels were higher in the ARDS group (all P < 0.05). Red blood cell count and hemoglobin did not differ significantly. Lymphocyte levels were lower in the ARDS group, although the difference was not statistically significant (P = 0.062). NLR, PLR, SII and SIRI were higher in the ARDS group (all P < 0.001), in **Table 1**. In a *post hoc* training-set analysis, SII had the highest direction-oriented univariable AUC (0.701), whereas all other variables had AUCs below 0.67 (**Supplementary Figure S2**). These analyses did not inform predictor selection.

**Table 1:** Baseline characteristics of mother–neonate pairs according to neonatal ARDS status.

Variables	Total (n=227)	Non-ARDS (n=120)	ARDS (n=107)	*P*
MA, years	31.00 [29.00, 34.00]	31.00 [28.80, 33.00]	32.00 [29.00, 36.00]	**0.032**
GA, weeks	35.70 [35.00, 36.00]	35.80 [35.00, 36.00]	35.60 [35.00, 36.10]	0.689
RBC count, ×10¹²/L	3.97 [3.71, 4.21]	4.00 [3.69, 4.22]	3.95 [3.77, 4.21]	0.900
WBC count, ×10^9^/L	8.40 [7.40, 10.20]	7.90 [6.90, 9.12]	8.90 [7.90, 10.70]	**<0.001**
PLT count, ×10^9^/L	201 [166, 240]	189 [154, 223]	212 [186, 263]	**<0.001**
Hb, g/L	121 [113, 130]	122 [114, 132]	119 [112, 127]	0.075
NEU, ×10^9^/L	6.17 [5.26, 7.64]	5.67 [4.99, 6.84]	6.91 [5.88, 8.32]	**<0.001**
LYM, ×10^9^/L	1.54 [1.25, 1.81]	1.56 [1.29, 1.88]	1.46 [1.21, 1.73]	0.062
MONO, ×10^9^/L	0.48 [0.40, 0.63]	0.46 [0.39, 0.56]	0.53 [0.42, 0.67]	**0.011**
NLR	4.14 [3.31, 5.18]	3.63 [2.99, 4.59]	4.72 [3.87, 5.84]	**<0.001**
PLR	134 [106, 168]	122 [97.6, 152]	147 [116, 193]	**<0.001**
SII	848 [598, 1140]	717 [533, 906]	1060 [767, 1340]	**<0.001**
SIRI	2.08 [1.47, 2.96]	1.76 [1.35, 2.33]	2.48 [1.89, 3.47]	**<0.001**
Sex (male)	124 (54.6%)	64 (53.3%)	60 (56.1%)	0.779
Low birth weight (Yes)	109 (48.0%)	62 (51.7%)	47 (43.9%)	0.302
ACS (Yes)	125 (55.1%)	82 (68.3%)	43 (40.2%)	**<0.001**
Gestational hypertension (Yes)	54 (23.8%)	32 (26.7%)	22 (20.6%)	0.356
GDM (Yes)	83 (36.6%)	39 (32.5%)	44 (41.1%)	0.227
ICP (Yes)	23 (10.1%)	10 (8.3%)	13 (12.1%)	0.465
Gestational hypothyroidism (Yes)	67 (29.5%)	30 (25.0%)	37 (34.6%)	0.152
PROM (Yes)	49 (21.6%)	22 (18.3%)	27 (25.2%)	0.271
CAM (Yes)	83 (36.6%)	39 (32.5%)	44 (41.1%)	0.227
PP (Yes)	66 (29.1%)	28 (23.3%)	38 (35.5%)	0.061
AI (Yes)	54 (23.8%)	14 (11.7%)	40 (37.4%)	**<0.001**

Values are median [interquartile range] for continuous variables and n (%) for categorical variables. P values are unadjusted two-group comparisons and are descriptive. Bold values indicate statistical significance. ARDS, acute respiratory distress syndrome; GA, gestational age; MA, maternal age; ACS, antenatal corticosteroids; GDM, gestational diabetes mellitus; ICP, intrahepatic cholestasis of pregnancy; PROM, premature rupture of membranes; CAM, chorioamnionitis; PP, placenta previa; AI, antenatal infection within 14 days before delivery; RBC, red blood cells; WBC, white blood cells; PLT, platelets; Hb, hemoglobin; NEU, neutrophils; LYM, lymphocytes; MONO, monocytes; NLR, neutrophil-to-lymphocyte ratio; PLR, platelet-to-lymphocyte ratio; SII, systemic immune-inflammation index; SIRI, systemic inflammation response index.

No values were missing for any of the 23 candidate predictors or for the outcome in the 227 analysed pairs, because neonates with incomplete clinical data had already been excluded at enrolment (**Figure 1**). Median imputation for continuous variables and most-frequent imputation for binary variables were nevertheless retained inside the modelling pipeline, so that they were refitted within each cross-validation fold and no information could pass between folds or between the training and internal test sets.

### Variable screening and final variable set

Bootstrap-stabilized elastic-net logistic regression initially identified eight stable predictors: AI, PLT, ACS, MA, GH, SII, NEU and GDM. Because SII is derived from PLT and NEU, retaining both the composite index and its components could duplicate inflammatory information and increase model complexity. Following clinical redundancy reduction, Group 1 retained SII and excluded PLT and NEU. The final set comprised AI, ACS, MA, GH, GDM and SII.

In repeated cross-validation, the six-predictor Group 1 set had a slightly higher mean AUC (0.729 ± 0.080) than Group 2 (0.718 ± 0.084) and Group 4 (0.708 ± 0.081), in **Table 2**. Group 1 also showed a favorable Brier score (0.212 ± 0.027) and specificity (0.733 ± 0.111). These numerical differences were descriptive. No non-inferiority margin or test was specified. The results supported use of the more parsimonious set that retained SII and excluded PLT and NEU.

**Table 2:** Random forest feature-set ablation in repeated cross-validation of the training set.

Feature set	Predictors	Predictors, n	AUC, mean ± SD	AP, mean ± SD	Brier, mean ± SD	Sensitivity, mean ± SD	Specificity, mean ± SD
Clinical plus inflammation	AI, ACS, MA, GH, GDM, SII	6	0.729 ± 0.080	0.724 ± 0.090	0.212 ± 0.027	0.567 ± 0.128	0.733 ± 0.111
Elastic net-selected full set	AI, PLT, ACS, MA, GH, SII, NEU, GDM	8	0.718 ± 0.084	0.741 ± 0.082	0.213 ± 0.026	0.544 ± 0.130	0.741 ± 0.110
Clinical plus original CBC	AI, ACS, MA, GH, GDM, PLT, NEU	7	0.708 ± 0.081	0.729 ± 0.078	0.215 ± 0.023	0.541 ± 0.135	0.760 ± 0.120
Clinical only	AI, ACS, MA, GH, GDM	5	0.697 ± 0.081	0.682 ± 0.083	0.223 ± 0.023	0.585 ± 0.115	0.708 ± 0.113

Feature-set ablation used random forest models. Results are from repeated stratified five-fold cross-validation with 20 repeats in the training set. The clinical-plus-inflammation set retained SII and excluded platelet and neutrophil counts to reduce redundancy. Comparisons are descriptive; no non-inferiority margin or test was specified. AUC, area under the receiver operating characteristic curve; AP, average precision; SD, standard deviation; CBC, complete blood count; MA, maternal age; ACS, antenatal corticosteroids; GDM, gestational diabetes mellitus; GH, gestational hypothyroidism; AI, antenatal infection within 14 days before delivery; PLT, platelets; NEU, neutrophils; SII, systemic immune-inflammation index.

### Model selection and development set comparison

After finalizing the six-predictor clinical-plus-inflammation set, we compared six algorithms using repeated stratified five-fold cross-validation with 20 repeats in the training set. These comprised logistic regression, elastic-net logistic regression, RF, GBM, XGBoost and SVM-RBF. Logistic regression and elastic-net logistic regression served as linear benchmarks. The complete four-feature-set by six-algorithm comparison is shown in **Supplementary Figure S3B**. The RF model using the final six-predictor set had the highest mean cross-validated AUC among the 24 combinations. **Table 3** reports results for RF and the two linear benchmarks using this feature set. Mean AUC was 0.729 ± 0.080 for RF, 0.706 ± 0.082 for logistic regression and 0.679 ± 0.089 for elastic-net logistic regression. The corresponding mean AP values were 0.724, 0.703 and 0.682, respectively. RF was therefore locked as the primary model on the basis of training-set cross-validation.

**Table 3:** Repeated cross-validation performance of the random forest and two linear benchmark models using the final six-predictor set in the training set.

Feature set	Model	Predictors, n	AUC, mean ± SD	AP, mean	Brier score, mean	Sensitivity, mean	Specificity, mean
Clinical plus inflammation	Random forest	6	0.729 ± 0.080	0.724	0.212	0.567	0.733
Clinical plus inflammation	Logistic regression	6	0.706 ± 0.082	0.703	0.220	0.525	0.744
Clinical plus inflammation	Elastic net logistic regression	6	0.679 ± 0.089	0.682	0.232	0.401	0.804

All models used the final clinical-plus-inflammation feature set (AI, ACS, MA, GH, GDM and SII). Results are means from repeated stratified five-fold cross-validation with 20 repeats in the training set. RF was selected using training-set cross-validation; internal test-set metrics did not enter the selection. AUC, area under the receiver operating characteristic curve; AP, average precision; SD, standard deviation; RF, random forest; AI, antenatal infection within 14 days before delivery; ACS, antenatal corticosteroids; MA, maternal age; GH, gestational hypothyroidism; GDM, gestational diabetes mellitus; SII, systemic immune-inflammation index.

### Discrimination and calibration in the internal test set

The final RF model’s discrimination, calibration and decision-curve results in the internal test set are shown in **Figure 2**. The corresponding point estimates and 95% confidence intervals are reported in **Table 4**.

**Figure 2:**
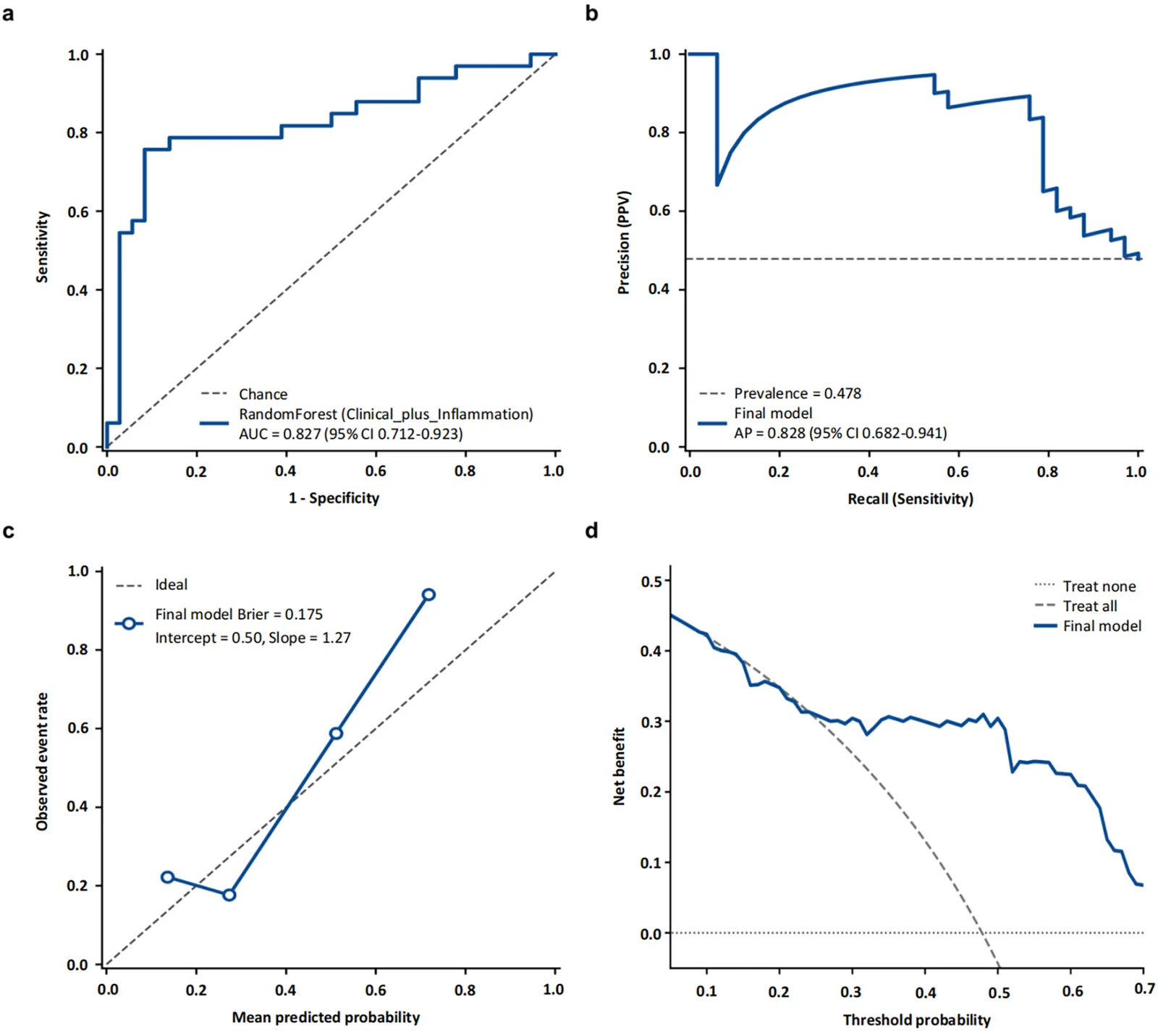
Predictive performance of the final random forest model in the internal test set. **(a)** Receiver operating characteristic curve. The dashed line indicates chance performance. **(b)** Precision–recall curve; the dashed horizontal line indicates ARDS prevalence in the internal test set. **(c)** Calibration plot based on four quantile-based risk groups among the 69 participants of the internal test set, so that each group contains approximately 17 participants; the dashed diagonal line indicates perfect calibration. Because the curve is derived from four grouped estimates in a small sample, it should be read as a coarse summary of calibration rather than a precise description of it. **(d)** Decision curve analysis comparing the final model with treat-all and treat-none strategies across threshold probabilities. AUC, area under the receiver operating characteristic curve; AP, average precision; ARDS, acute respiratory distress syndrome; CI, confidence interval.

**Table 4:** Internal test performance and training-set bootstrap optimism correction for the locked random forest model.

Metric	Estimate	95% CI/description
Feature set	Clinical plus inflammation	AI, ACS, MA, GH, GDM, and SII
Model	Random forest	Primary machine-learning model
AUC	0.827	0.712–0.923
AP	0.828	0.682–0.941
Brier score	0.175	0.130–0.223
Calibration intercept	0.501	−0.057 to 1.110
Calibration slope	1.269	0.735–2.291
Apparent AUC in training set	0.889	
Mean optimism for AUC	0.096	
Optimism-corrected AUC	0.793	
Optimism-corrected Brier score	0.199	

The internal test set included 69 participants and 33 ARDS events. AUC, AP and Brier-score point estimates were calculated in the original internal test set; percentile confidence intervals were obtained from 1,000 bootstrap resamples of that set. Calibration intercept and slope have ideal values of 0 and 1, respectively, and their confidence intervals were obtained from the same 1,000 bootstrap resamples; both intervals include their ideal value. The calibration curve underlying these estimates is based on four quantile groups among the 69 participants of the internal test set. Apparent and optimism-corrected estimates refer to the locked six-predictor random forest pipeline in the training set and were obtained using 500 bootstrap resamples. Predictor screening, feature-set construction and algorithm selection were not repeated within these resamples. The training-set cross-validated AUC of 0.729 is reported as the principal internal performance estimate; see Results. ARDS, acute respiratory distress syndrome; AP, average precision; AUC, area under the receiver operating characteristic curve; CI, confidence interval.

In the internal test set, the RF model achieved an AUC of 0.827 (95% CI, 0.712–0.923) and an AP of 0.828 (95% CI, 0.682–0.941), in **Figures 2a, b**. The Brier score was 0.175 (95% CI, 0.130–0.223). The calibration intercept was 0.501 (95% CI, −0.057 – 1.110) and the calibration slope was 1.269 (95% CI, 0.735–2.291) (**Figure 2c**). The positive intercept indicates a systematic offset in which predicted probabilities were on average lower than observed event rates, corresponding to a shift of approximately 0.50 on the logit scale relative to the ideal value of 0. The slope above 1 indicates that predicted risks were less extreme than observed risks. Both confidence intervals included their ideal values, so the size of these departures cannot be established at this sample size. This imprecision is, however, distinct from the systematic offset that the intercept describes. The calibration curve was based on four quantile groups among 69 participants, approximately 17 per group, and is correspondingly coarse. Decision curve analysis showed net benefit relative to the default strategies over portions of the evaluated threshold range, but the curves crossed and did not establish uniform clinical superiority (**Figure 2d**). **Table 4** provides the corresponding estimates and confidence intervals.

### Internal validation and overfitting assessment

For the fixed RF pipeline, the apparent training-set AUC was 0.889 and estimated optimism was 0.096, yielding an optimism-corrected AUC of 0.793. The optimism-corrected Brier score was 0.199 (**Table 4**). These results indicate optimism in apparent training performance. Because predictor screening, feature-set construction and algorithm selection were not repeated within bootstrap samples, the correction does not capture uncertainty across the full development process.

Three estimates of discrimination are therefore available for this model, and they are not interchangeable. The training-set cross-validated AUC was 0.729 ± 0.080, the fixed-pipeline optimism-corrected AUC was 0.793, and the internal test-set AUC was 0.827 (95% CI, 0.712–0.923). The internal test-set value exceeded the cross-validated value by 0.098 and the optimism-corrected value by 0.034. The three differ in what each one includes. The cross-validated estimate repeats model fitting within the training partition but holds the six predictors fixed. The optimism-corrected estimate starts from the apparent training-set AUC of 0.889. It did not repeat predictor screening, feature-set construction or algorithm selection within its resamples. The internal test partition had been accessed during earlier exploratory evaluation and was therefore not a strictly untouched holdout. None of the three values is simultaneously independent of prior exposure and corrected for the full development process. We designate the training-set cross-validated AUC of 0.729 as the principal internal performance estimate, because it was computed entirely within the development partition and did not directly use the previously accessed internal test set. We report the other two as secondary estimates. That choice carries its own limitation: predictor screening was performed once in the full training set before cross-validation. Therefore, 0.729 does not propagate the uncertainty introduced by that step and may itself remain optimistic relative to a fully nested estimate. The confidence interval of the internal test-set estimate (0.712–0.923) includes both of the other two values, so the observed ordering is compatible with sampling variation and no single explanation for it can be established from these data.

### Threshold selection and clinical classification performance

At the training-derived Youden threshold of 0.424, sensitivity was 0.788, specificity was 0.778, PPV was 0.765 and NPV was 0.800. Accuracy was 0.783 and the F1 score was 0.776 (**Figures 3a, b**; **Supplementary Table S1**). Sensitivity-oriented and fixed 0.50 thresholds were also reported to show alternative trade-offs between sensitivity and specificity. PPV, NPV, accuracy and the F1 score are conditional on the ARDS proportion of this cohort, which was 47.1% overall (107/227) and 47.8% in the internal test set (33/69). The same dependence applies to the calibration curve in **Figure 2c**. Sensitivity and specificity do not depend on this proportion. These predictive values should therefore not be read as transportable absolute risks, and would differ in settings where the proportion of ARDS among ventilated neonates with respiratory distress differs from that observed here.

**Figure 3:**
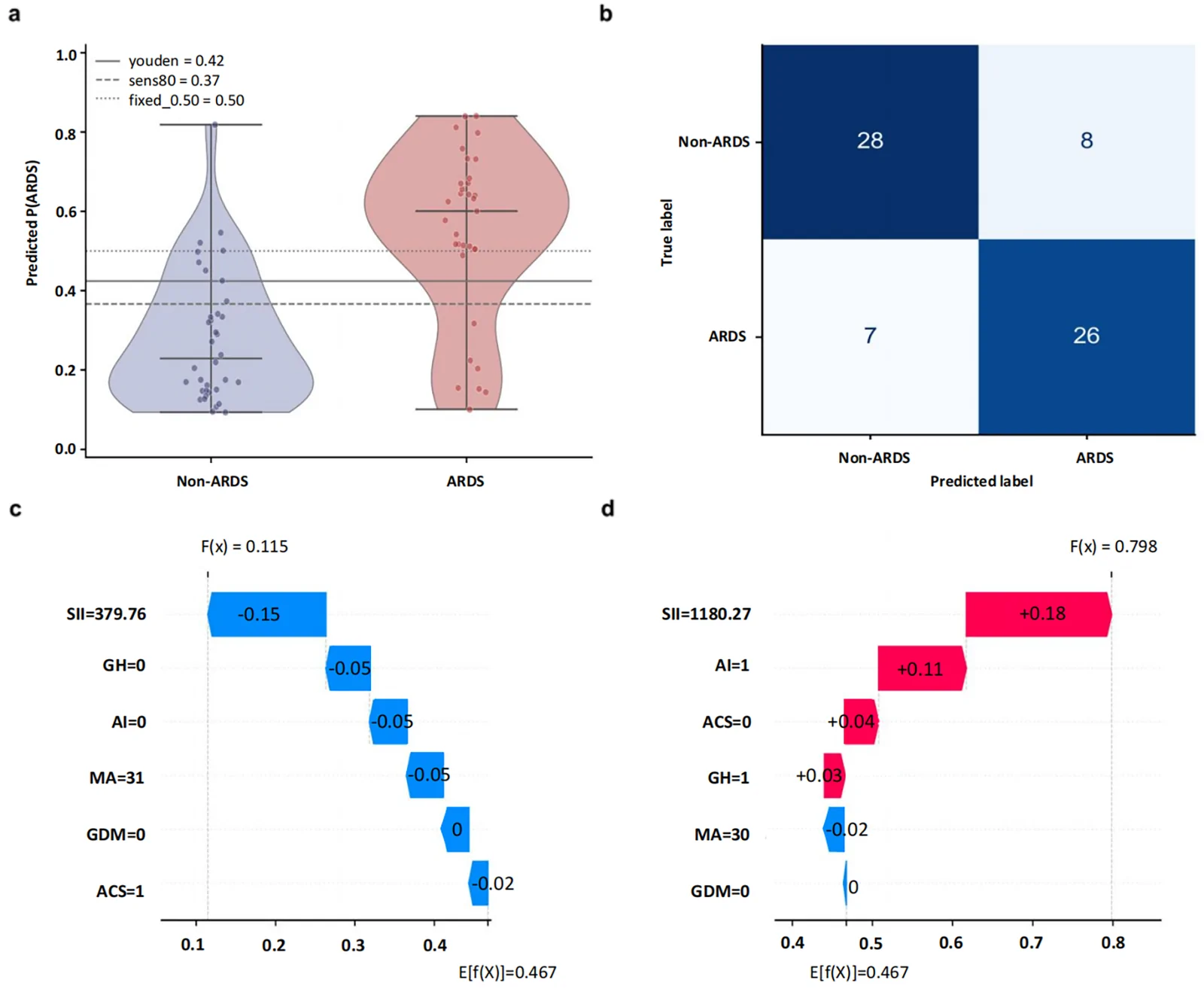
Clinical presentation and individual-level explanations of the final model in the internal test set. **(a)** Distribution of predicted ARDS probabilities by observed outcome; horizontal lines indicate the training-derived Youden, sensitivity-oriented and fixed 0.50 thresholds. **(b)** Confusion matrix at the training-derived Youden threshold. **(c)** SHAP waterfall plot for an illustrative correctly classified low-risk non-ARDS case. **(d)** SHAP waterfall plot for an illustrative correctly classified high-risk ARDS case. E[f(X)] denotes the baseline predicted probability and f(x) denotes the individual predicted probability. Blue features decrease and red features increase the predicted probability of ARDS. AI, antenatal infection within 14 days before delivery; GH, gestational hypothyroidism; ACS, antenatal corticosteroids; GDM, gestational diabetes mellitus.

### Model interpretation

We used SHAP to examine global and individual model behavior (**Figures 3c, d**, **4**). SII had the largest global contribution to predicted ARDS risk, followed by AI, MA, ACS, GH and GDM. SII showed a nonlinear marginal association with model-predicted risk, which generally increased at higher SII values. This descriptive analysis did not infer any diagnostic threshold. The waterfall plots illustrate the contribution of each predictor in individual cases. In the low-risk case, low SII made the largest negative contribution and reduced the model output to 0.115. In the high-risk case, elevated SII and antenatal infection made the largest positive contributions. The absence of ACS use and the presence of gestational hypothyroidism contributed more modestly, whereas maternal age contributed slightly negatively, yielding a predicted probability of 0.798. These case-specific SHAP values describe model output and do not establish biological interaction or causal synergy. The ranking of mean absolute SHAP values was descriptively similar in the development cohort (**Supplementary Figure S4**). Because SII is calculated from the same blood-cell counts as the other inflammatory indices and remains correlated with them, its rank in this analysis reflects its contribution within the six-predictor set that was carried forward, and not an independent or specific biological role. Had a correlated index such as NLR or PLR been retained in its place, part of the attributed contribution would have transferred to that variable. SHAP quantifies how each input moves the output of this particular model but it does not identify which underlying biological quantity is responsible.

**Figure 4:**
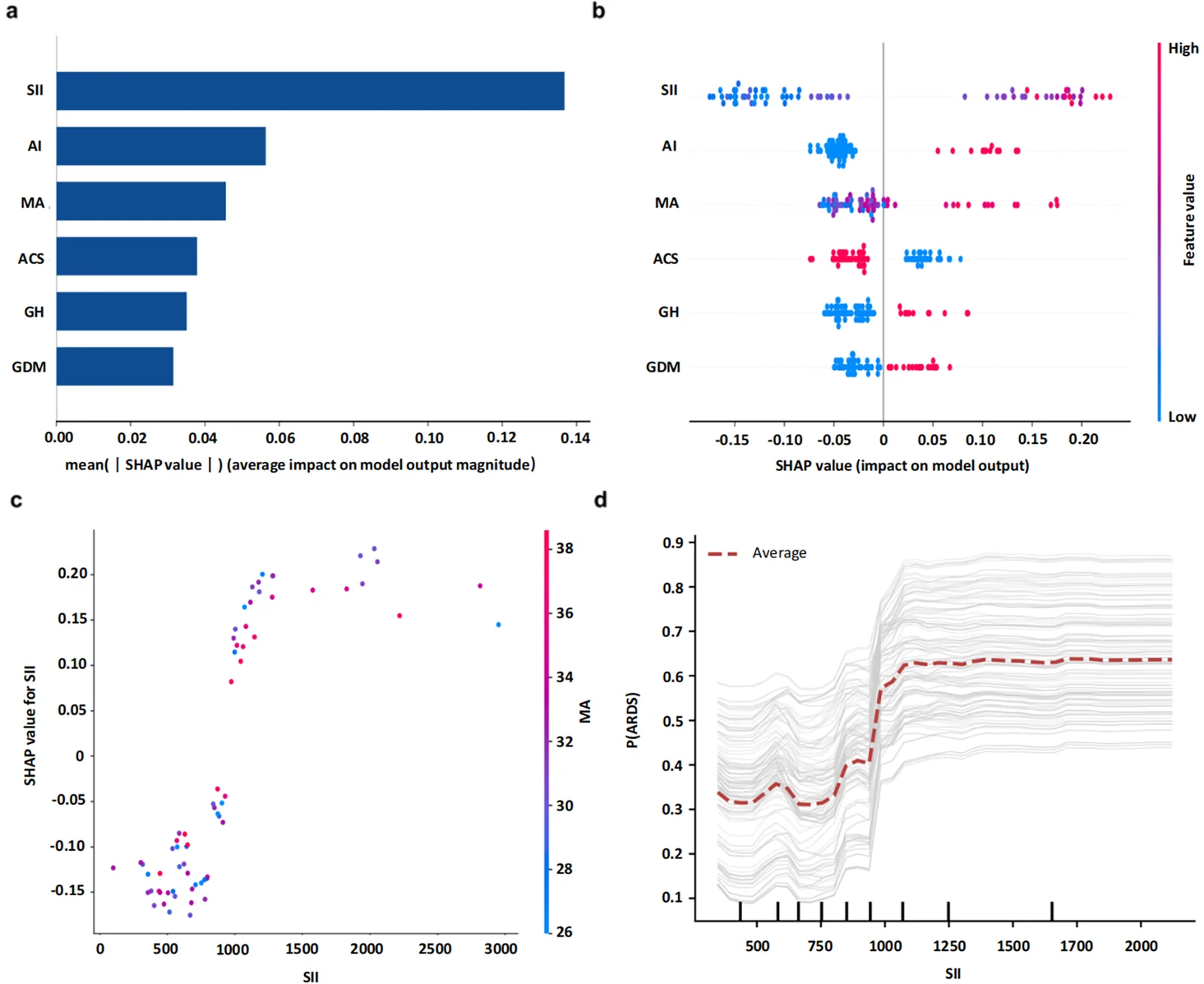
Global and feature-specific interpretation of the final random forest model. **(a)** Mean absolute SHAP values showing global feature importance. **(b)** SHAP beeswarm plot showing the distribution and direction of feature contributions to predicted ARDS risk; red indicates higher feature values and blue indicates lower feature values. For binary variables, red corresponds to presence and blue to absence. **(c)** SHAP dependence plot for SII, with point color indicating maternal age. **(d)** Partial dependence curve (red dashed line) and individual conditional expectation curves (grey lines) for SII. **(a–c)** use the internal test set (n = 69); **(d)** uses the training set (n = 158). SHAP values quantify feature contributions to model output and should not be interpreted as causal effects. SII, systemic immune-inflammation index; ARDS, acute respiratory distress syndrome; MA, maternal age; AI, antenatal infection within 14 days before delivery; GH, gestational hypothyroidism; ACS, antenatal corticosteroids; GDM, gestational diabetes mellitus.

### *Post hoc* ACS sensitivity analysis

In the *post hoc* sensitivity analysis, median SII remained higher in the ARDS group within both ACS strata. Among ACS recipients, median SII was 892 (IQR, 613–1,146) in the ARDS group and 713 (IQR, 523–881) in the non-ARDS group (P = 0.008). The corresponding values among participants without documented ACS use were 1,117 (IQR, 940–1,604) and 725 (IQR, 546–937), respectively (P < 0.001). In the full-cohort adjusted model, each 1-SD increase in log2-transformed SII was associated with higher odds of ARDS (OR = 1.82, 95%CI, 1.28–2.58, P < 0.001). Adding the SII-by-ACS interaction provided no statistical evidence that this association differed according to ACS use (interaction OR = 0.58, 95%CI, 0.28–1.20, P = 0.140; **Supplementary Table S4**). These exploratory analyses were not used for model development or performance evaluation.

### Benchmark model comparison and primary model selection

We compared the final RF model with logistic regression and elastic-net logistic regression after RF had been selected from training-set cross-validation (**Supplementary Figure S5**; **Supplementary Tables S2, 3**). Logistic regression had a numerically higher internal-test-set AUC than RF (0.838 versus 0.827). The paired difference was not statistically definitive (ΔAUC = −0.011, 95% CI, −0.079–0.062, P = 0.744, **Supplementary Figure S3A**). RF had a lower Brier score and a calibration slope closer to 1 in this partition, whereas the decision curves crossed across thresholds. These post-selection comparisons were descriptive and did not determine primary-model selection.

To demonstrate model implementation, we developed a research-use web interface for the locked RF model (**Figure 5**). The intended point of use in the clinical pathway is after a late-preterm or term neonate has developed respiratory distress requiring mechanical ventilation. The model is not intended, and has not been evaluated, for antenatal screening of unselected pregnancies, and it cannot be applied before that eligibility is known. Because recorded ACS use is one of its inputs, it must also not be used to guide the initial decision to administer antenatal corticosteroids. Users enter the six predictors and receive a model-estimated probability together with a SHAP-based explanation. The displayed value is conditional on the case mix of this development cohort, is not a calibrated absolute risk for other populations, and the interface has not undergone prospective clinical-impact evaluation.

**Figure 5:**
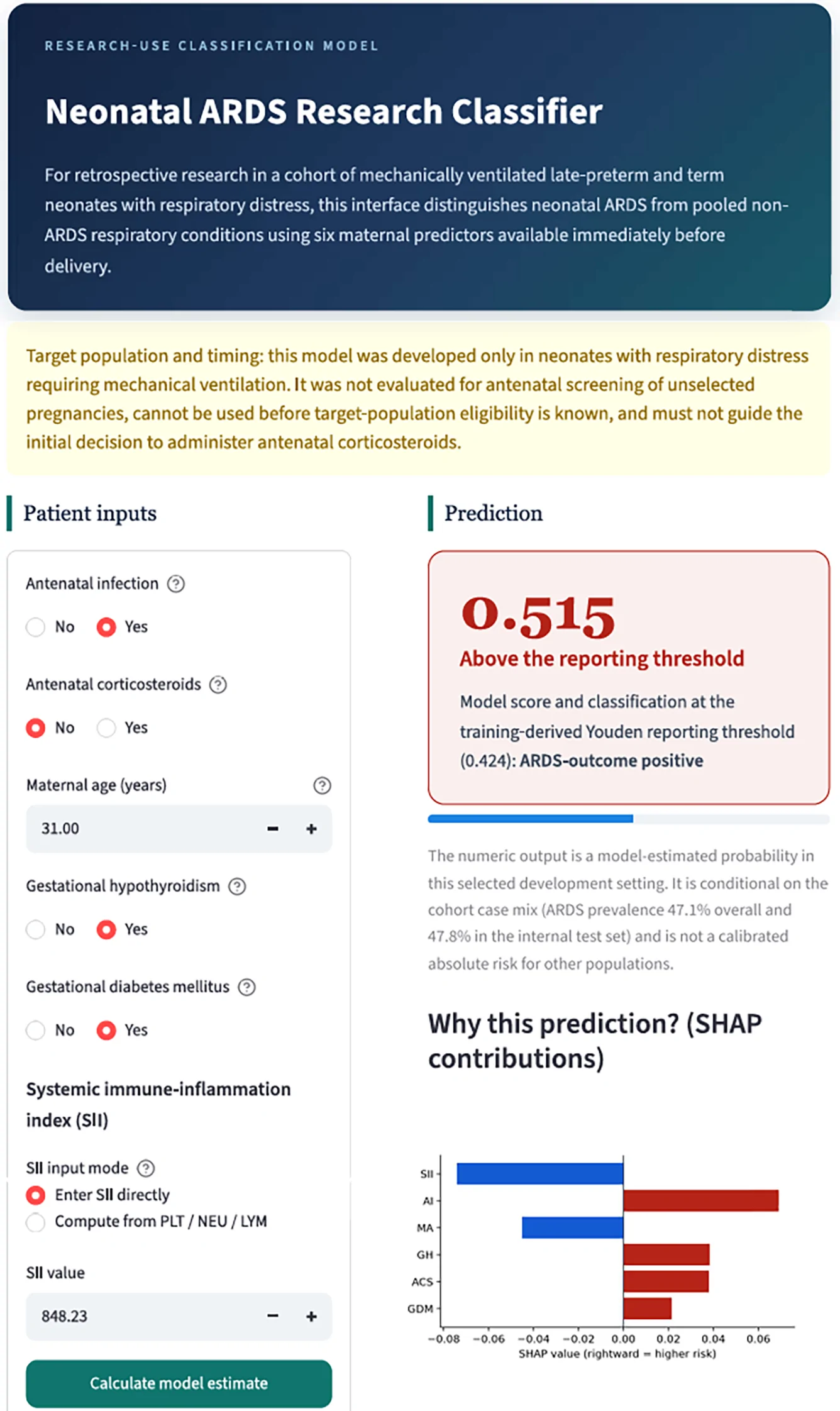
Screenshot of the research‑use web interface for the locked random forest model. The interface accepts the six predictors of the final model and returns a model‑estimated probability together with a SHAP‑based explanation, and classifies that probability at the training‑derived Youden reporting threshold of 0.424. It is intended for use only after a late‑preterm or term neonate has developed respiratory distress requiring mechanical ventilation, and not for antenatal screening of unselected pregnancies. The displayed probability is conditional on the case mix of this development cohort and is not a calibrated absolute risk for other populations; the displayed classification is not a validated clinical risk category. The interface has not undergone prospective clinical‑impact evaluation. SHAP, Shapley additive explanations; Antenatal infection within 14 days before delivery.

## Discussion

This study used data from 227 mother–infant pairs involving mechanically ventilated late-preterm and term neonates with respiratory distress to evaluate six machine learning algorithms for discriminating neonatal ARDS from non-ARDS respiratory conditions. The final RF model used maternal SII, AI, MA, GH, GDM and recorded ACS use. These variables were prediction inputs rather than independent causal effects. SHAP ranked SII as the largest contributor and showed a nonlinear association with model-predicted risk. The findings apply to this selected high-risk cohort and do not establish performance in unselected pregnancies or neonates who do not require mechanical ventilation.

Composite inflammatory indices combine several innate immune-cell components, and the predictive performance of SII in this study was consistent with previous reports (20). One hypothesis consistent with these associations is that maternal systemic inflammation and immune imbalance are involved in the pathways leading to neonatal ARDS, although the present retrospective design cannot test that hypothesis. Excessive neutrophil recruitment and activation are central to ARDS pathogenesis. Activated neutrophils damage the alveolar epithelium and capillary endothelium through reactive oxygen species, proteolytic enzymes and neutrophil extracellular traps. These processes increase alveolar–capillary permeability and promote pulmonary edema (25–27). Neutrophil recruitment is driven largely by inflammatory mediators and chemokines released from endothelial and immune cells. This cascade is amplified in ARDS, leading to more severe local injury and impaired lung function (28). Monocytes also contribute to ARDS pathogenesis. Following recruitment into lung tissue, circulating monocytes differentiate into inflammatory macrophages. Together with resident alveolar macrophages, these cells regulate neutrophil‑mediated lung injury (5, 29). Their activation and infiltration contribute to alveolar epithelial and vascular endothelial damage. Lymphopenia may reflect reduced immune regulation and further aggravate uncontrolled inflammation (28). While these mechanisms offer mechanistic context for the present study, they were not directly evaluated using this maternal blood‑count model.

Platelet and neutrophil counts differed between outcome groups and were initially retained by the bootstrap-stabilized elastic net. They were removed during clinical redundancy reduction because SII already incorporates both measures, and they are not separate predictors in the final model. Platelets have recognized inflammatory and immune-thrombotic functions (30), including roles in innate immune regulation (31) and platelet–neutrophil interactions (32). In the feature-set analysis, the clinical-plus-inflammation set had the highest mean cross-validated AUC and used fewer variables than the larger screened sets. Within this model, SII provided a parsimonious summary of inflammatory information without an apparent loss of cross-validated discrimination. However, the numerical differences were modest and descriptive, and did not establish that SII should replace its component blood counts in clinical practice.

Antenatal infection documented within 14 days before delivery was more frequent in the ARDS group and contributed positively to RF output. Literature on intrauterine inflammation describes possible effects on fetal immunity and lung development (33, 34), competing maturational and injurious effects (33, 35), and inflammatory priming before postnatal insults (34). That evidence cannot be assumed to characterize the broader infection variable used here. The study did not record infection site, pathogen, severity, treatment or fetal inflammatory response, so a specific intrauterine mechanism cannot be inferred.

Recorded ACS use contributed negatively to model output and was inversely associated with ARDS in this cohort. The complete blood counts used to calculate SII preceded ACS initiation by at least 72 h, making an acute corticosteroid-induced change in the measured blood-cell counts an unlikely explanation for the SII association. Corticosteroids have anti-inflammatory actions (36) and promote fetal lung maturation and surfactant production (37). Observational studies have also examined associations between antenatal exposure and pulmonary outcomes (38). In the *post hoc* sensitivity analysis, SII remained associated with ARDS after adjustment for ACS and the other model covariates, and there was no statistical evidence that the association differed according to ACS use. However, ACS was administered according to clinical indications rather than randomly assigned. Confounding by indication, gestational timing, treatment regimen and other unmeasured differences therefore remain possible.

Maternal age was slightly higher in the ARDS group and ranked third by mean absolute SHAP value. In the *post hoc* adjusted sensitivity model, higher maternal age remained associated with ARDS. Earlier work has linked maternal age to neonatal morbidity (39) and adverse perinatal outcomes (40, 41), with proposed placental, vascular and inflammatory pathways (41, 42). This is in line with the background against which the findings of this study should be interpreted.

Bootstrap-stabilized elastic net retained GH and GDM as obstetric comorbidity predictors. Neither differed significantly between outcome groups at baseline. Maternal SII and SIRI during pregnancy have nonetheless been associated with gestational diabetes mellitus (43), so the co-occurrence of GDM with inflammatory indices has an existing basis in the literature. Their inclusion indicates that they contributed to multivariable model predictions, but this finding does not establish their independent or causal effects. Their predictive contribution and clinical relevance should be assessed in independent cohorts with more detailed exposure measurement.

We screened predictors using 1,000 bootstrap samples and compared six algorithms by repeated five-fold cross-validation in the training set. RF achieved the highest mean cross-validated AUC and was therefore locked as the primary model before final internal test-set assessment. The fixed-pipeline bootstrap yielded an optimism-corrected training-set AUC of 0.793 but did not repeat predictor screening, feature-set construction or algorithm selection. The internal test-set DCA was descriptive and did not demonstrate uniform clinical superiority or an ability to reduce overtreatment in practice.

In the internal test set, logistic regression had a numerically higher AUC than RF, and the paired difference was not statistically definitive. RF had a lower Brier score and a calibration slope closer to 1 in this partition, but the decision curves crossed across thresholds. These findings did not establish uniform superiority of RF, and calibration was itself not satisfactory. The calibration intercept of 0.501 pointed to systematic offset, with predicted probabilities averaging lower than observed event rates, and the slope of 1.269 indicates that predicted risks were less extreme than observed risks. Both confidence intervals included their ideal values. Therefore, this sample size could not establish either departure. However, interval width did not rule out a systematic offset of this direction and magnitude. Such an offset required correction before predicted probabilities could be applied to individual patients. This was directly relevant here, because the model is presented through a web interface that returns an individual probability, so recalibration in the target setting is a prerequisite for any such use. Maternal inflammatory indicators showed nonlinear patterns in model output, these descriptive patterns did not establish clinical thresholds, biological interactions or causal synergy. SHAP improved transparency by describing feature contributions, but it did not convert predictions into independent or causal effects.

The RF was locked as the primary model because it achieved the highest mean cross-validated AUC among the 24 feature-set by algorithm combinations evaluated in the training set (0.729 ± 0.080 versus 0.706 ± 0.082 for logistic regression). On the evidence available here, we do not claim that the additional complexity was rewarded. The two models did not differ definitively in internal test-set discrimination, and the cross-validated difference was small relative to its variability. For a six-predictor model in a cohort of this size, a logistic regression would be simpler to report, to inspect and to recalibrate. So we have provided its full coefficients and standardization constants (**Supplementary Table S7**) so that readers can apply and evaluate it directly. Whether a nonlinear model offers any advantage in this setting is a question that only a larger, externally validated comparison can answer.

The three discrimination estimates should likewise not be read as interchangeable. The internal test-set AUC of 0.827 was higher than both the training-set cross-validated estimate of 0.729 and the fixed-pipeline optimism-corrected estimate of 0.793. Several factors bear on how this ordering should be read, and they cannot be separated in a study of this size. The confidence interval of the internal test-set estimate spans both of the other values, so sampling variation could account for the observed ordering. The internal test partition had also been accessed during earlier exploratory evaluation, the optimism correction was not nested over the full development process, and the two partitions differed in case mix, including for two predictors of the final model (**Supplementary Table S5**). Therefore, we treat 0.729 as the principal internal estimate and 0.793 and 0.827 as secondary estimates, none of which represents independent external performance, rather than attributing the ordering to any one of these causes.

This study has additional important limitations. First, the retrospective cohort was drawn from only two hospitals and had no temporal or external validation, so performance estimates may be optimistic. Second, the sample size of the internal test subset was modest, comprising merely 69 neonates with 33 ARDS events. This produced wide confidence intervals for discrimination and calibration metrics, limiting the precision of internal performance assessment. Third, the eligibility criteria restricted the cohort to neonates born at 34–40 weeks who developed respiratory distress within 7 days and required mechanical ventilation. Neonates below 34 weeks, and those with respiratory distress that did not require ventilation, were not represented. The findings cannot be extrapolated to them. because eligibility is established only once ventilation has begun, the cohort is also selected on a post-natal event, so the reported performance describes discrimination within this selected population rather than antenatal prediction in an unselected one. Fourth, the fixed-pipeline bootstrap did not propagate uncertainty from predictor screening or model selection. Fifth, ACS use was not randomized and remained susceptible to confounding by indication, gestational timing and treatment regimen. Although the CBC preceded ACS initiation by at least 72 h and the adjusted analysis found no statistical evidence of an SII-by-ACS interaction, these observations do not eliminate residual confounding or establish an ACS treatment effect. Because ACS is prescribed on the basis of gestational age, the perceived imminence of preterm birth and obstetric indication, and because the interval between administration and delivery and the completeness of the course were not recorded, the contribution of recorded ACS use to model output should be read as a marker of the clinical circumstances in which it was prescribed rather than as a treatment effect. The sensitivity analysis combined the training and test partitions after model development and assessed association only, it was not independent validation. Sixth, the infection variable lacked information on site, pathogen, severity and treatment, and the blood-count indices did not capture specific cytokine or lung-injury pathways. Finally, this study should therefore be regarded as exploratory. Before deploying any web‑based calculator or bedside tool, the model must be evaluated in cohorts that are geographically and temporally separate from the original development dataset. Agreement between predicted probabilities and observed outcomes should be directly assessed. Recalibration should be performed if required. Furthermore, the model should be explicitly compared against a simple logistic‑regression model built using the same set of predictors.

## Data Availability

The datasets presented in this study can be found in online repositories. The names of the repository/repositories and accession number(s) can be found in the article/**Supplementary Material**.
