# Supplementary material for: A predictive model based on maternal immune-inflammatory markers for ARDS identification in mechanically ventilated late-preterm and term neonates
Source: Front Immunol. 2026 Oct 6;17:1889452. doi: 10.3389/fimmu.2026.1889452 (PMC13680245; doi:10.3389/fimmu.2026.1889452)
Supplement: Supplementary file 1 [file DataSheet1.pdf]

Supplementary Material

Predictive value of maternal prenatal blood immune-inflammatory markers for neonatal acute respiratory distress syndrome based on machine learning

Appendix 1. The Montreux definition of neonatal ARDS

**Characteristics**	**Details**
Time frame	Acute onset (i.e., within one week) from a known or suspected clinical insult.
Exclusion criteria	RDS, TTN, or congenital anomalies as a primary current acute respiratory condition.
Lung imaging	Diffuse, bilateral, and irregular opacities or infiltrates, or complete opacification of the lungs, which are not fully explained by local effusions, atelectasis, RDS, TTN, or congenital anomalies.
Origin of oedema	Absence of congenital heart disease explaining the oedema (this includes ductus arteriosus with pulmonary overflow if no acute pulmonary haemorrhage exists). Echocardiography is needed to verify the origin of oedema.
Oxygenation deficit expressed as OI	Mild ARDS: $4 \leq OI < 8$;<br>Moderate ARDS: $8 \leq OI < 16$;<br>Severe ARDS: $OI \geq 16$

ARDS, acute respiratory distress syndrome; RDS, respiratory distress syndrome; TTN, transient tachypnoea of the neonate; OI, oxygenation index.

Appendix 2. Identification of ARDS, RDS, TTN, and infectious pneumonia

Characteristics	ARDS	RDS	TTN	Infectious pneumonia
GA	No thresholds	The younger the GA, the higher the incidence	No thresholds	No thresholds
Birth weight	No thresholds	The lower the weight, the higher the incidence	—	—
Maternal disease	Severity	Mildness	—	Maternal chorioamnionitis, GBS colonization, intrapartum fever and so on.
Physiopathology	Diffuse injury of alveolar capillaries	Primary surfactant deficiency	Delayed absorption or excessive accumulation of fetal lung fluid	Pathogen invasion of lung parenchyma with inflammatory infiltration
Response to antenatal corticosteroids	Low response	Good response	—	—
Pulmonary injury	Alveolar cavity, pulmonary vessels and interstitial inflammation cell infiltration, scattered hyaline membrane	No or little alveolar cavity, pulmonary vessels and interstitial inflammation cell infiltration, symmetrically hyaline membrane	Interstitial edema, no hyaline membrane	Patchy or diffuse consolidation, bronchopneumonia pattern, may have pleural effusion
Bronchoalveolar lavage fluid	High concentration of inflammatory factors and proteins	Low or normal concentration of pro-inflammatory factors and proteins	Normal or elevated protein content	Pathogen positive (culture, PCR, or mNGS), elevated inflammatory markers
Inflammatory response	Common	Rare	Rare	Common
Onset features	Gradually aggravating, no significant remission within 3 days	Gradually aggravating, gradual remission within 3–6 days	Appearing within 24 h after birth, resolving within 72 h	Early-onset: within 48 h after birth; Late-onset: after 1 week of life
Other organ injury	Common	Rare	Rare	Possible (sepsis, shock, DIC)
X-ray	Diffuse, bilateral, and irregular opacities or infiltrates, or complete opacification of the lungs, which are not fully explained by local effusions, atelectasis, RDS, TTN, or congenital anomalies	Reticulogranular pattern ("ground glass"), air bronchograms, even complete opacification or "white lung"	Hilar engorgement, prominent vascular markings, fissural thickening, no alveolar opacities	Patchy or diffuse parenchymal infiltrates, air bronchograms, pleural effusion; changes persist > 48 h
Lung ultrasound	—	Homogeneous loss of aeration with diffuse alveolar pattern ("white lung"), irregular pleural line with subpleural consolidations, LUS 10–12	Heterogenous interstitial pattern alternating with at least one normal zone (A-pattern), no consolidations, LUS 4–6	Lung consolidation with air bronchograms or fluid bronchograms, pleural line abnormality, B-lines in non-consolidated areas
Response to PS	Mild or moderate effect	Remarkable effect	No effect	No effect (unless coexisting RDS)
PS times and dose	Multiple times, large dose	One or two times, small dose	Not indicated	Not indicated

GA, gestational age; ARDS, acute respiratory distress syndrome; RDS, respiratory distress syndrome; TTN, transient tachypnoea of the neonate; GBS, group B Streptococcus; PCR, polymerase chain reaction; mNGS, metagenomic next-generation sequencing; DIC, disseminated intravascular coagulation; PS, pulmonary surfactant; LUS, lung ultrasound score.

Appendix 3. Definition of pregnancy-related comorbidities

Diagnosis	Standard definition
Gestational diabetes mellitus (GDM)	Glucose intolerance first recognized during pregnancy (usually 24–28 weeks) by an oral glucose tolerance test (OGTT) meeting or exceeding thresholds: fasting ≥ 5.1 mmol/L, 1 h ≥ 10.0 mmol/L, or 2 h ≥ 8.5 mmol/L.
Gestational hypertension (CHT)	New-onset hypertension after 20 weeks of gestation without proteinuria, defined as systolic blood pressure ≥ 140 mmHg and/or diastolic blood pressure ≥ 90 mmHg on at least two occasions 4 hours apart.
Pregnancy complicated by chorioamnionitis (CAM)	Intra-amniotic infection, typically diagnosed clinically by maternal fever (≥ 38.0 °C) plus two or more of: maternal tachycardia, fetal tachycardia, uterine tenderness, purulent amniotic fluid, or maternal leukocytosis.
Pregnancy complicated by hypothyroidism (GH)	Elevated thyroid-stimulating hormone (TSH) beyond trimester-specific reference ranges, with or without decreased free thyroxine (FT4), diagnosed during pregnancy.
Placenta previa (PP)	Placenta fully or partially covering the internal cervical os, diagnosed by transvaginal ultrasound after 16–20 weeks of gestation (typically confirmed in the third trimester).
Premature rupture of membranes (PROM)	Rupture of the amniotic sac before the onset of labor, confirmed by sterile speculum examination showing amniotic fluid pooling from the cervical os, positive nitrazine test, or fern test.
Antenatal infection within 14 days before delivery (AI)	Documented maternal infection requiring medical attention occurring within 14 days before delivery, including but not limited to respiratory, urinary, genital tract, or systemic infections.
Antenatal corticosteroid use (ACS)	Administration of corticosteroids (e.g., betamethasone or dexamethasone) to the mother prior to delivery to accelerate fetal lung maturation, typically indicated for threatened preterm birth between 24 and 34 weeks of gestation.
Intrahepatic cholestasis of pregnancy (ICP)	Pruritus of pregnancy with elevated fasting serum total bile acid concentration, in the absence of other hepatobiliary disease, with resolution after delivery.

Appendix 4. Candidate predictors considered for model development

Dataset code	Predictor	Type or unit
Gender	Neonatal sex	Binary; male = 1
ACS	Antenatal corticosteroid use	Binary
CHT	Gestational hypertension	Binary
GDM	Gestational diabetes mellitus	Binary
ICP	Intrahepatic cholestasis of pregnancy	Binary
GH	Gestational hypothyroidism	Binary
PROM	Premature rupture of membranes	Binary
CAM	Chorioamnionitis	Binary
PP	Placenta previa	Binary
AI	Antenatal infection within 14 days before delivery	Binary
MA	Maternal age	Years
GA	Gestational age at delivery	Weeks
RBC	Red blood cell count	$\times 10^{12}$/L
WBC	White blood cell count	$\times 10^{9}$/L
PLT	Platelet count	$\times 10^{9}$/L
Hb	Haemoglobin	g/L
NEU	Neutrophil count	$\times 10^{9}$/L
LYM	Lymphocyte count	$\times 10^{9}$/L
MONO	Monocyte count	$\times 10^{9}$/L
NLR	Neutrophil-to-lymphocyte ratio	Derived: NEU/LYM
PLR	Platelet-to-lymphocyte ratio	Derived: PLT/LYM
SII	Systemic immune-inflammation index	Derived: PLT × NEU/LYM
SIRI	Systemic inflammation response index	Derived: NEU × MONO/LYM

The dataset contained 24 non-outcome fields. Low birth weight was excluded a priori before screening because it is determined after birth, leaving 23 eligible candidate predictors. Dataset codes are retained here for reproducibility; CHT denotes gestational hypertension and GH denotes gestational hypothyroidism in this dataset. The intended model-use time point was before delivery after recorded ACS status was ascertainable, rather than at the earlier complete-blood-count sampling time. ACS, antenatal corticosteroid; CHT, gestational hypertension; GDM, gestational diabetes mellitus; ICP, intrahepatic cholestasis of pregnancy; GH, gestational hypothyroidism; PROM, premature rupture of membranes; CAM, chorioamnionitis; PP, placenta previa; AI, antenatal infection within 14 days before delivery; MA, maternal age; GA, gestational age; RBC, red blood cells; WBC, white blood cells; PLT, platelets; Hb, haemoglobin; NEU, neutrophils; LYM, lymphocytes; MONO, monocytes; NLR, neutrophil-to-lymphocyte ratio; PLR, platelet-to-lymphocyte ratio; SII, systemic immune-inflammation index; SIRI, systemic inflammation response index.

Supplementary Figures

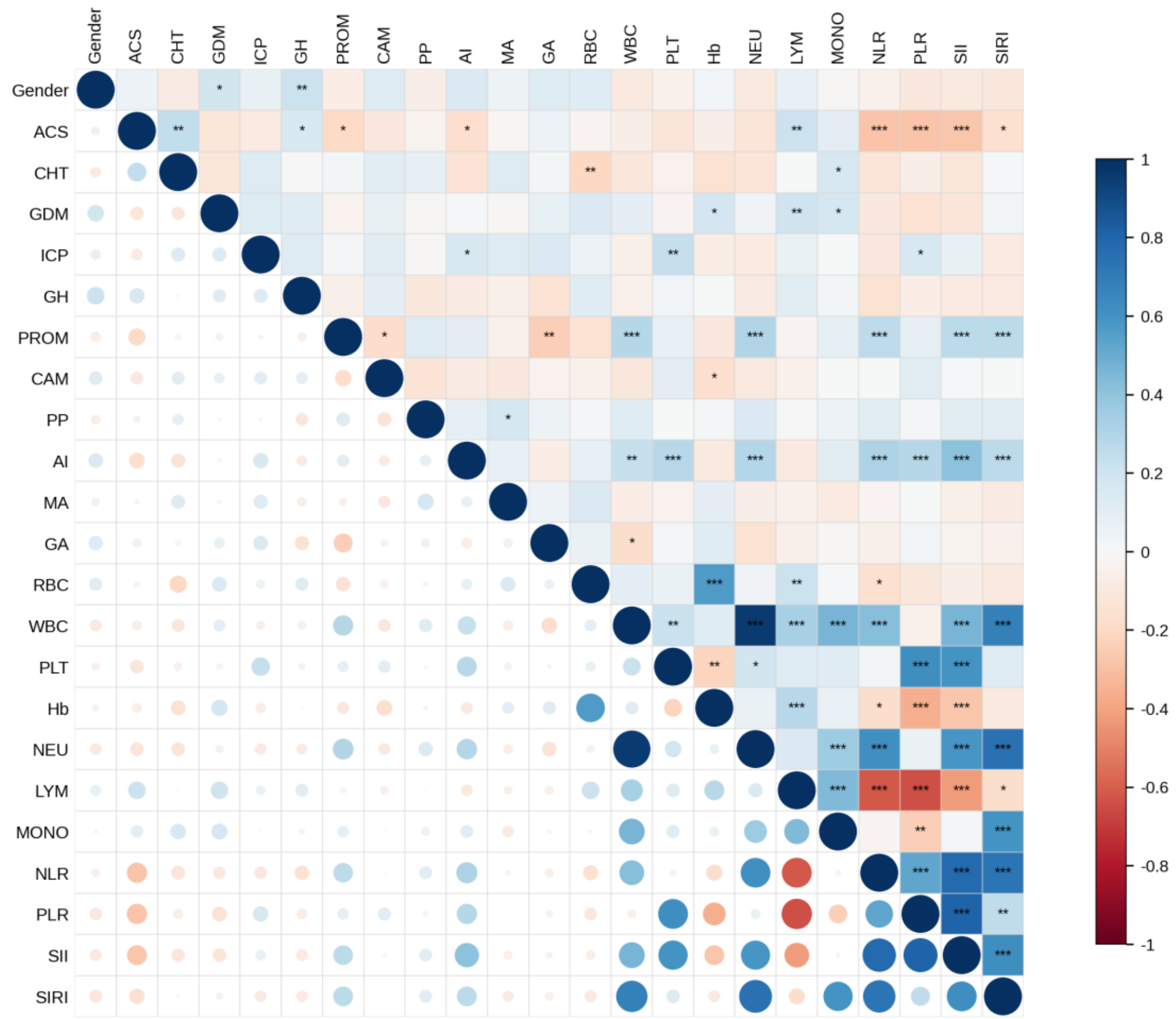

Supplementary Figure S1. Spearman correlation matrix of the 23 candidate predictors in the training set (n = 158). The upper triangle shows colour-coded correlation coefficients with significance markers; the lower triangle shows the same coefficients as circles scaled by the absolute value of the coefficient. Blue indicates positive and red negative correlation. *P < 0.05, **P < 0.01, ***P < 0.001. Low birth weight was excluded a priori from the screening pool because it is a postnatal variable, and is therefore not shown. This matrix is descriptive and is presented to document the correlation structure among candidate predictors that motivated the use of bootstrap-stabilised elastic net for predictor screening; it was not itself used to include or exclude any predictor. Gender, neonatal sex; ACS, antenatal corticosteroids; CHT, gestational hypertension; GDM, gestational diabetes mellitus; ICP, intrahepatic cholestasis of pregnancy; GH, gestational hypothyroidism; PROM, premature rupture of membranes; CAM, chorioamnionitis; PP, placenta previa; AI, antenatal infection within 14 days before delivery; MA, maternal age; GA, gestational age; RBC, red blood cells; WBC, white blood cells; PLT, platelets; Hb, haemoglobin; NEU, neutrophils; LYM, lymphocytes; MONO, monocytes; NLR, neutrophil-to-lymphocyte ratio; PLR, platelet-to-lymphocyte ratio; SII, systemic immune-inflammation index; SIRI, systemic inflammation response index.

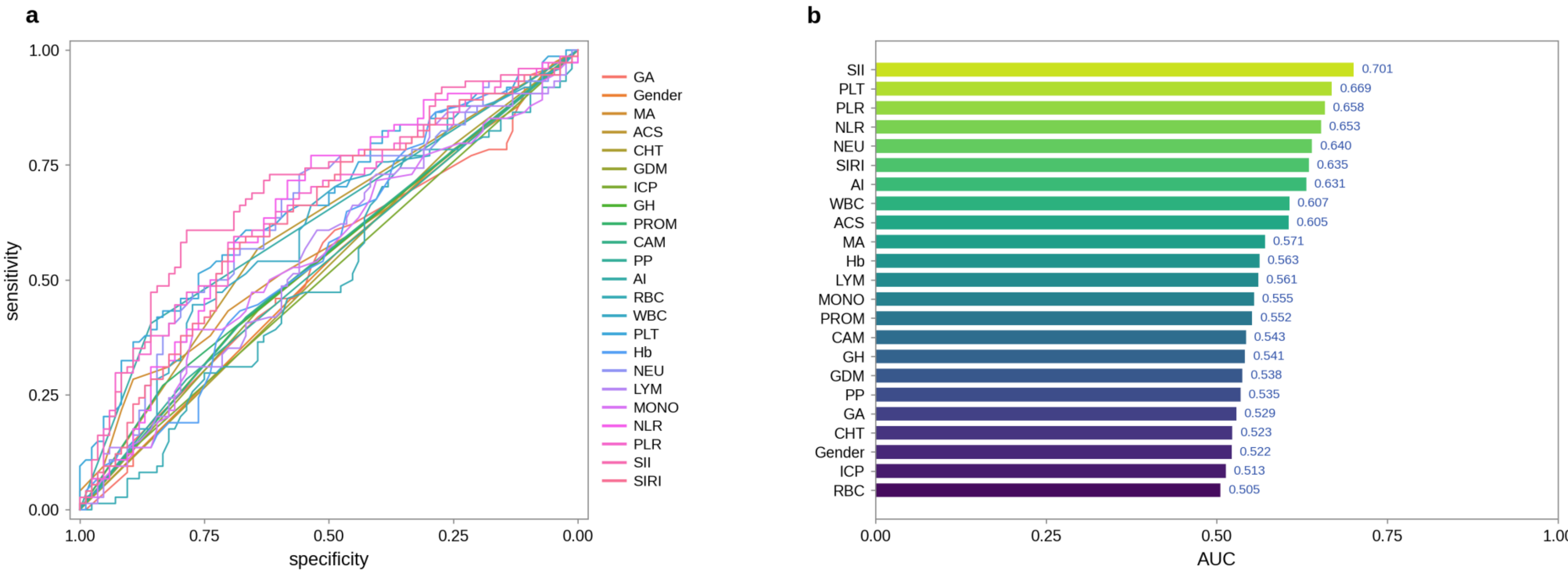

Supplementary Figure S2. Post hoc univariable discrimination of the 23 candidate predictors for neonatal ARDS in the training set (n = 158). (a) Receiver operating characteristic curves for each candidate variable. (b) Direction-oriented area under the curve (AUC) for each variable, ranked in descending order. Variables inversely associated with ARDS were oriented so that the reported AUC exceeds 0.50; these were ACS, Hb, LYM, GA and CHT. Direction-oriented AUC summarises the strength of discrimination only and does not indicate the direction of association with ARDS. Low birth weight was excluded a priori from the screening pool and is not shown. These analyses are descriptive and were performed after model development; they did not inform predictor selection, which was performed by bootstrap-stabilised elastic net logistic regression in the training set only. Abbreviations as in Supplementary Figure S1.

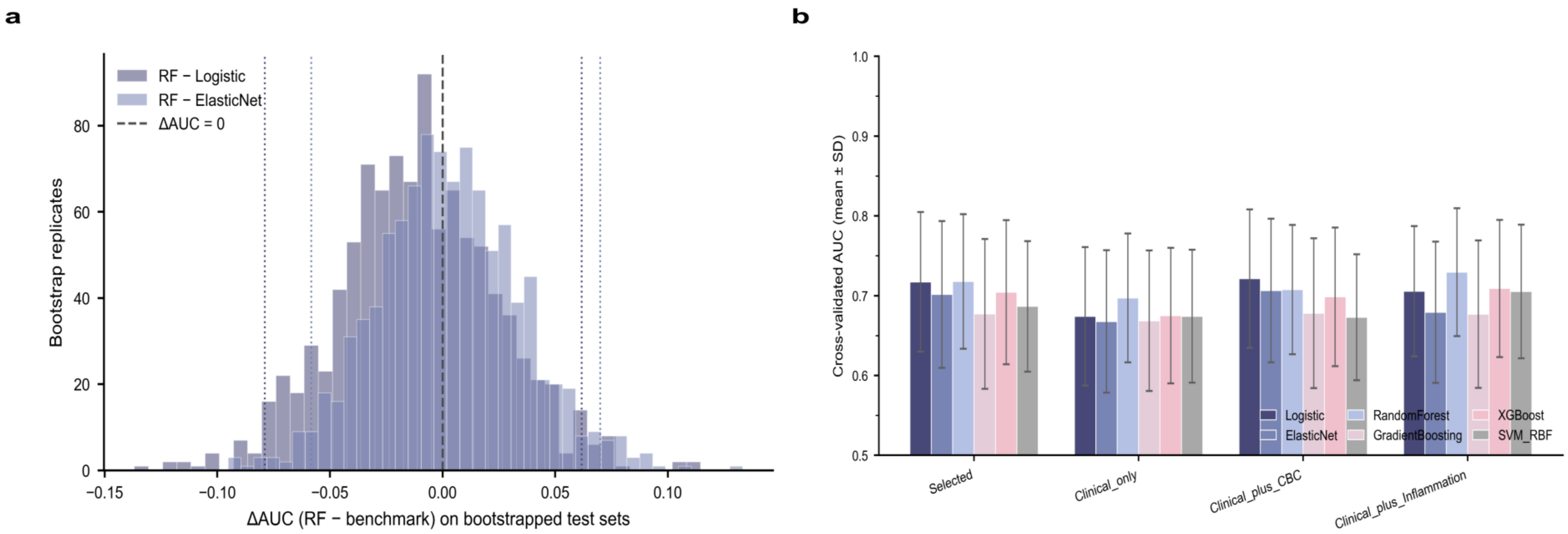

Supplementary Figure S3. Supplementary analyses of model comparison. The two panels are based on different cohorts. (a) Internal test set (n = 69): distribution of the difference in AUC (ΔAUC) between the random forest model and each linear benchmark across 1,000 paired bootstrap resamples; dotted lines mark the 95% percentile interval and the dashed line marks ΔAUC = 0. (b) Training set (n = 158): mean ± SD cross-validated AUC for all four candidate feature sets and all six candidate algorithms under repeated stratified five-fold cross-validation (20 repeats). The random forest model using the Clinical_plus_Inflammation feature set achieved the highest mean cross-validated AUC (0.729 ± 0.080) among the 24 feature-set × algorithm combinations and was selected from training-set results; internal-test-set comparisons did not enter primary-model selection.

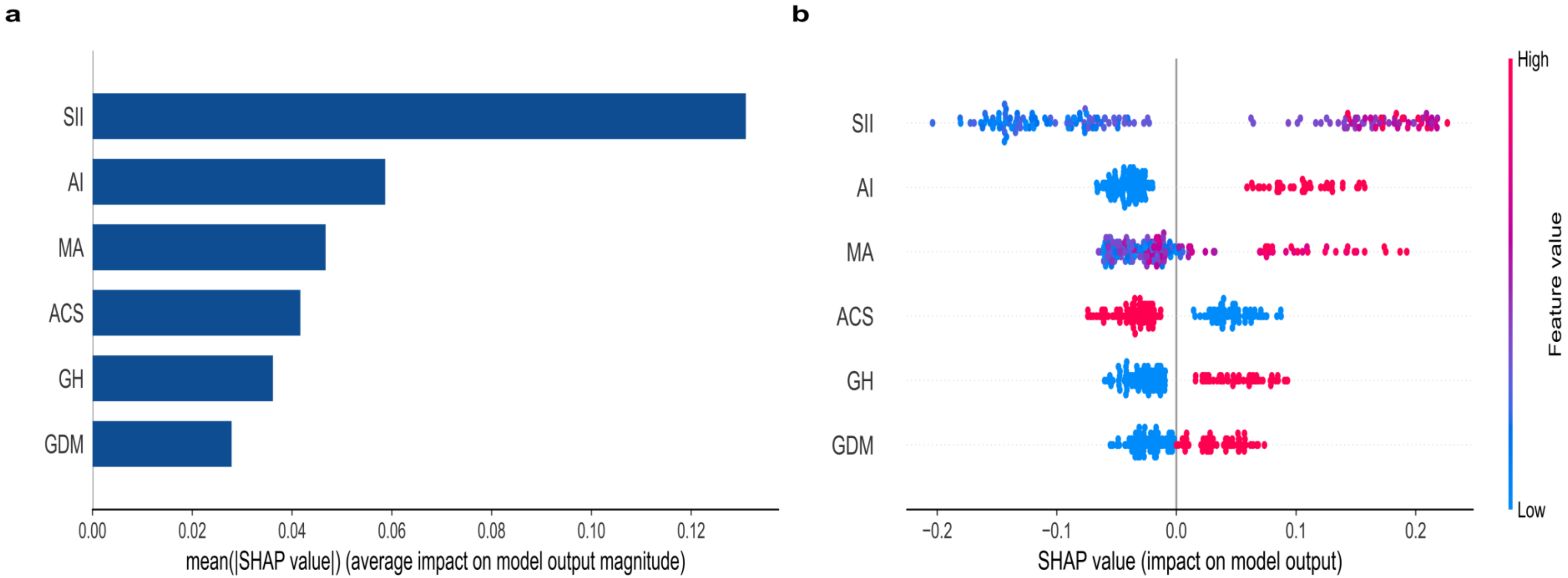

Supplementary Figure S4. SHAP-based global interpretation of the final random forest model in the development cohort. (a) Mean absolute SHAP value for each predictor. (b) Beeswarm plot of SHAP values, coloured by feature value. The rank order of predictor importance (SII > AI > MA > ACS > GH > GDM) is descriptively similar to that obtained in the internal test set (Figure 3a,b). SII, systemic immune-inflammation index; AI, antenatal infection within 14 days before delivery; MA, maternal age; ACS, antenatal corticosteroids; GH, gestational hypothyroidism; GDM, gestational diabetes mellitus.

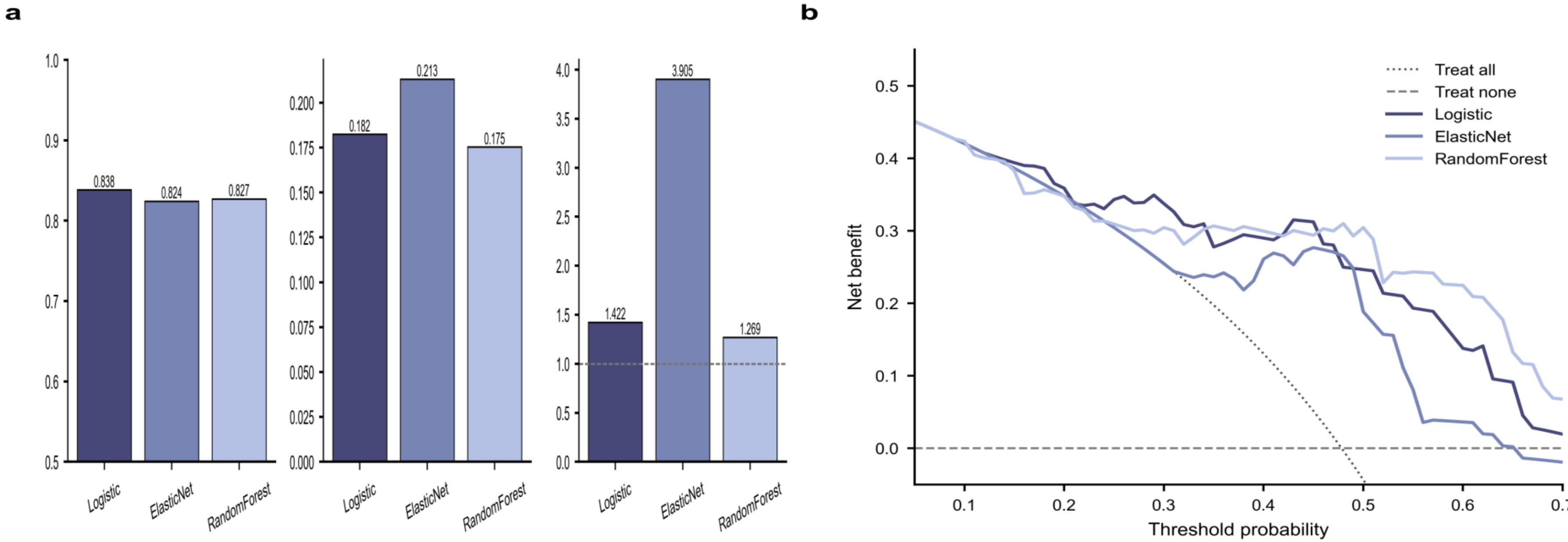

Supplementary Figure S5. Head-to-head comparison of the final random forest model with the two linear benchmark models in the internal test set (n = 69). All three models were fitted on the same six predictors. (a) Test-set AUC, Brier score and calibration slope for logistic regression, elastic net logistic regression and random forest; the dashed line in the right panel marks the ideal calibration slope of 1. (b) Decision curves for the three models against the treat-all and treat-none strategies; the curves cross at several thresholds, so no model was uniformly superior across the threshold range. Paired bootstrap comparisons did not show a statistically definitive difference in AUC between the random forest and either benchmark (Supplementary Table S3). In this single internal test-set partition the random forest showed the lowest Brier score, the calibration slope closest to 1, and the highest mean net benefit; these are descriptive observations and were not used to select the primary model.

Supplementary Tables

Supplementary Table S1. Classification performance of the final random forest model in the internal test set (n = 69) at probability thresholds derived from the training set.

Threshold strategy	Cut-off	Sensitivity	Specificity	PPV	NPV	Accuracy	F1	TP	FP	TN	FN
Youden index (primary)	0.424	0.788	0.778	0.765	0.800	0.783	0.776	26	8	28	7
Sensitivity-oriented (≥ 0.80)	0.367	0.788	0.750	0.743	0.794	0.768	0.765	26	9	27	7
Sensitivity-oriented (≥ 0.85)	0.319	0.788	0.611	0.650	0.759	0.696	0.712	26	14	22	7
Fixed 0.50	0.500	0.758	0.889	0.862	0.800	0.826	0.806	25	4	32	8

All thresholds were derived from out-of-fold predicted probabilities in the training set and then applied unchanged to the internal test set; the values shown are those achieved in the test set. The Youden-index threshold was adopted as the primary operating point for reporting. Because predicted probabilities are discrete, the two sensitivity-oriented thresholds achieved the same test-set sensitivity as the Youden threshold. PPV, positive predictive value; NPV, negative predictive value; TP, true positive; FP, false positive; TN, true negative; FN, false negative.

Supplementary Table S2. Performance of the final random forest model and the two linear benchmark models in the internal test set (n = 69).

Model	CV mean AUC	CV mean Brier	Test AUC (95% CI)	Test AP (95% CI)	Test Brier (95% CI)	Calibration intercept	Calibration slope
Logistic regression	0.706	0.220	0.838 (0.727–0.929)	0.795 (0.631–0.933)	0.182 (0.141–0.226)	0.491	1.422
Elastic net logistic regression	0.679	0.232	0.824 (0.714–0.918)	0.782 (0.620–0.932)	0.213 (0.194–0.234)	0.678	3.905
Random forest (final model)	0.729	0.212	0.827 (0.712–0.923)	0.828 (0.682–0.941)	0.175 (0.130–0.223)	0.501	1.269

All point estimates were computed on the original internal test set; percentile confidence intervals were derived from 1,000 bootstrap resamples of that test set. CV, cross-validation (repeated stratified five-fold, 20 repeats, training set); AUC, area under the receiver operating characteristic curve; AP, average precision (a summary of the precision–recall curve); CI, confidence interval. The AP point estimate for the random forest model (0.828) is the value reported in Table 4 and Figure 2b of the main text. The mean decision-curve net benefit across the threshold range was 0.254 for logistic regression, 0.213 for elastic net logistic regression and 0.267 for random forest. All three models were fitted on the same six predictors; the random forest was locked as the primary model on the basis of training-set cross-validation alone.

Supplementary Table S3. Paired bootstrap comparison of internal-test-set AUC between the final random forest model and each linear benchmark model.

Comparison	AUC (random forest)	AUC (benchmark)	ΔAUC	95% CI of ΔAUC	P value	Bootstrap replicates
Random forest − logistic regression	0.827	0.838	−0.011	(−0.079 to 0.062)	0.744	1,000
Random forest − elastic net logistic regression	0.827	0.824	0.003	(−0.058 to 0.070)	0.962	1,000

ΔAUC was computed as the AUC of the random forest model minus the AUC of the benchmark model within each of 1,000 paired bootstrap resamples of the internal test set. Two-sided P values were derived from the proportion of resamples in which the sign of ΔAUC was reversed. Neither comparison reached statistical significance, indicating that the random forest model did not demonstrate a discriminative advantage over the linear benchmark models in this sample.

Supplementary Table S4. Post hoc sensitivity analysis of the association between maternal SII and neonatal ARDS according to recorded ACS use.

a. SII distribution and univariable discrimination within ACS strata

ACS stratum	n (ARDS/non-ARDS)	SII in ARDS, median [IQR]	SII in non-ARDS, median [IQR]	P value	SII AUC
ACS use	125 (43/82)	892 [613–1,146]	713 [523–881]	0.008	0.644
No ACS use	102 (64/38)	1,117 [940–1,604]	725 [546–937]	<0.001	0.756

b. Multivariable logistic-regression sensitivity models

Term	Model 1 OR (95% CI)	P value	Model 2 OR (95% CI)	P value
$\log_2$(SII), per 1 SD	1.82 (1.28–2.58)	<0.001	2.50 (1.40–4.46)	0.002
ACS use	0.40 (0.21–0.73)	0.003	0.40 (0.21–0.74)	0.004
Antenatal infection	2.90 (1.36–6.18)	0.006	2.83 (1.32–6.07)	0.007
Maternal age, per year	1.12 (1.03–1.21)	0.005	1.11 (1.03–1.20)	0.007
Gestational hypothyroidism	2.05 (1.05–3.99)	0.035	2.01 (1.03–3.90)	0.041
Gestational diabetes mellitus	1.25 (0.67–2.33)	0.480	1.33 (0.71–2.50)	0.369
$\log_2$(SII) × ACS	—	—	0.58 (0.28–1.20)	0.140

This was a post hoc exploratory full-cohort analysis conducted after model development and combined the training and internal test partitions. It was not used for predictor screening, feature-set construction, algorithm selection, threshold selection or model-performance evaluation and should not be interpreted as independent validation. Panel a reports two-sided Mann–Whitney U tests and univariable SII AUC within each ACS stratum. In panel b, SII was log2-transformed and then standardized across the full cohort; its OR is per 1-SD increase. Model 1 included SII, ACS, antenatal infection, maternal age, gestational hypothyroidism and gestational diabetes mellitus. Model 2 added the SII-by-ACS interaction term. Maternal-age ORs are per year. These observational associations do not establish an ACS treatment effect. ACS, antenatal corticosteroid; ARDS, acute respiratory distress syndrome; AUC, area under the receiver operating characteristic curve; CI, confidence interval; IQR, interquartile range; OR, odds ratio; SII, systemic immune-inflammation index.

Supplementary Table S5. Baseline balance between the training set and the internal test set.

Variable	Training set (n = 158)	Internal test set (n = 69)	SMD
Neonatal ARDS (outcome)	74 (46.8)	33 (47.8)	−0.020
Neonatal sex, male (Gender)	86 (54.4)	38 (55.1)	−0.013
Antenatal corticosteroid use (ACS)	86 (54.4)	39 (56.5)	−0.042
Gestational hypertension (CHT)	38 (24.1)	16 (23.2)	0.020
Gestational diabetes mellitus (GDM)	62 (39.2)	21 (30.4)	0.186
Intrahepatic cholestasis of pregnancy (ICP)	17 (10.8)	6 (8.7)	0.070
Gestational hypothyroidism (GH)	55 (34.8)	12 (17.4)	0.405
Premature rupture of membranes (PROM)	34 (21.5)	15 (21.7)	−0.005
Chorioamnionitis (CAM)	59 (37.3)	24 (34.8)	0.053
Placenta previa (PP)	39 (24.7)	27 (39.1)	−0.314
Antenatal infection (AI)	42 (26.6)	12 (17.4)	0.223
Maternal age, years (MA)	31.71 (4.11)	32.04 (3.88)	−0.084
Gestational age, weeks (GA)	35.75 (1.22)	35.79 (1.45)	−0.035
Red blood cells, $\times 10^{12}$/L (RBC)	3.99 (0.43)	3.92 (0.39)	0.167
White blood cells, $\times 10^{9}$/L (WBC)	8.75 (2.17)	8.94 (2.41)	−0.084
Platelets, $\times 10^{9}$/L (PLT)	210.02 (60.30)	196.19 (52.80)	0.244
Haemoglobin, g/L (Hb)	121.52 (13.22)	120.14 (13.36)	0.103
Neutrophils, $\times 10^{9}$/L (NEU)	6.58 (2.02)	6.70 (2.36)	−0.056
Lymphocytes, $\times 10^{9}$/L (LYM)	1.65 (1.49)	1.58 (0.58)	0.068
Monocytes, $\times 10^{9}$/L (MONO)	0.52 (0.19)	0.53 (0.16)	−0.039
Neutrophil-to-lymphocyte ratio (NLR)	4.75 (2.91)	4.86 (2.76)	−0.039
Platelet-to-lymphocyte ratio (PLR)	149.66 (79.55)	138.48 (60.74)	0.158
Systemic immune-inflammation index (SII)	1013.91 (817.88)	945.73 (562.25)	0.097
Systemic inflammation response index (SIRI)	2.34 (1.27)	2.55 (1.62)	−0.143

Binary variables are reported as n (%) and continuous variables as mean (SD). The standardized mean difference (SMD) was calculated as the difference in means, or in proportions, divided by the pooled standard deviation; positive values indicate a higher value in the training set. No significance testing was performed, because the partitions were created by stratified random sampling on the outcome and any difference between them arises by chance. Stratification kept the outcome closely balanced (SMD −0.020). For interpretation of the remaining values, the standard error of an SMD at these partition sizes is approximately 0.144, so under chance alone approximately 11 of the 23 candidate predictors would be expected to exceed the conventional threshold of 0.10; 9 did so. That threshold is conventionally applied to residual imbalance after matching or weighting and is not a criterion for a single random split. The partitions nevertheless differ in case mix, and two of the variables showing the larger differences, gestational hypothyroidism (34.8% versus 17.4%, SMD 0.405) and antenatal infection (26.6% versus 17.4%, SMD 0.223), are predictors in the final model. Estimates obtained in the internal test set therefore reflect the case mix of that partition and should not be read as estimates of transportable performance.

Supplementary Table S6. Finalized configurations of the six candidate algorithms.

Algorithm	Continuous predictors standardized	Configuration and hyperparameters
Logistic regression	Yes	L2 penalty (scikit-learn default C = 1.0); solver, liblinear; maximum iterations, 5,000; no class weighting
Elastic-net logistic regression	Yes	penalty, elasticnet; solver, saga; internally tuned by 5-fold cross-validation within the training folds over l1_ratio ∈ {0.1, 0.3, 0.5, 0.7, 0.9, 1.0} and 20 values of C on a log scale from 10^{-3} to 10^{1}, selected by ROC-AUC; maximum iterations, 10,000; refitted on the full inner training data
Random forest (final model)	No	n_estimators, 500; max_depth, none; min_samples_leaf, 5; max_features, sqrt; bootstrap resampling with default settings
Gradient boosting machine	No	n_estimators, 150; learning_rate, 0.03; max_depth, 2; remaining scikit-learn defaults
XGBoost	No	n_estimators, 200; max_depth, 2; learning_rate, 0.03; subsample, 0.8; colsample_bytree, 0.8; reg_lambda, 2.0; reg_alpha, 0.0; eval_metric, logloss; random_state, 42; n_jobs, −1; remaining parameters at the defaults of the xgboost implementation
Support vector machine, RBF kernel	Yes	kernel, rbf; C, 1.0; gamma, scale; probability estimates enabled by Platt scaling

All algorithms were implemented in scikit-learn, except XGBoost, which used the xgboost package. A fixed random seed of 42 was used throughout. Imputation, standardization where applicable, and model fitting were encapsulated in a single pipeline and refitted within each cross-validation fold. Hyperparameters were fixed a priori for all algorithms except elastic-net logistic regression, whose penalty parameters were tuned by an inner cross-validation loop nested within the training folds; no hyperparameter was tuned using the internal test set.

Supplementary Table S7. Coefficients of the six-predictor logistic regression fitted in the training set.

Term	Coefficient (β)	Odds ratio	Scaling applied before the model
Intercept	−0.3565	—	—
Maternal age, years (MA)	+0.3871	1.473	(value − 31.709) / 4.093
Systemic immune-inflammation index (SII)	+0.6933	2.000	(value − 1013.912) / 815.290
Antenatal infection (AI)	+0.9090	2.482	0 = no, 1 = yes
Antenatal corticosteroid use (ACS)	−0.5781	0.561	0 = no, 1 = yes
Gestational hypothyroidism (GH)	+0.6463	1.908	0 = no, 1 = yes
Gestational diabetes mellitus (GDM)	+0.3156	1.371	0 = no, 1 = yes

This model is the logistic-regression benchmark reported in Supplementary Tables S2 and S3; it is not the primary model of the study, which is the random forest. It is provided so that readers can apply and evaluate a usable closed form of a six-predictor model without running code. The predicted probability is obtained as $p = 1 / (1 + \exp(-\eta))$, where η is the intercept plus the sum of each coefficient multiplied by its scaled predictor value; the two continuous predictors must first be scaled using the training-set constants given in the final column. Coefficients are on the scaled predictor scale, so each odds ratio for a continuous predictor corresponds to a one-standard-deviation increase. In the internal test set this model achieved an AUC of 0.838 (95% CI, 0.727–0.929). Its coefficients describe the fitted prediction model and are not estimates of independent or causal effects.
